# Gradient-Nested Organic/Inorganic Aerogels Achieve High Mechanical Strength and Feedback-Tunable Microwave Absorption

**DOI:** 10.34133/research.1074

**Published:** 2026-01-14

**Authors:** Xiao Liu, Zhike Si, Yongxin Qian, Lihong Wu, Xuefei Xu, Gengping Wan, Guizhen Wang

**Affiliations:** ^1^ Institute of Electromagnetic Protection Materials and Spectral Innovation Technology, State Key Laboratory of Tropic Ocean Engineering Materials and Materials Evaluation, School of Materials Science and Engineering, Hainan University, Haikou, Hainan 570228, China.; ^2^ Center for New Pharmaceutical Development and Testing of Haikou, Center for Advanced Studies in Precision Instruments, Haikou, Hainan 570228, China.; ^3^ Key Laboratory of Pico Electron Microscopy of Hainan Province, Center for Advanced Studies in Precision Instruments, Haikou, Hainan 570228, China.

## Abstract

The integration of multiband and multimode systems has substantially increased the complexity of electromagnetic interference, revealing critical limitations of existing absorbers in tuning efficiency and structural resilience. Here, we propose a nested microaerogel strategy to simultaneously improve the mechanical strength and microwave absorption (MA) performance of polyimide (PI) aerogel. By incorporating pomelo-peel-derived cellulose micronetworks and helical carbon nanocoil microaerogels to construct a gradient-nested framework, the long-standing issues of filler agglomeration and structural shrinkage in aerogels are effectively resolved. The aerogel combines low density, impact resistance, and thermal insulation with remarkable MA performance. The compressive strength reaches 1.3 MPa, which is about 20 times higher than pristine PI aerogel. Furthermore, it exhibits a minimum reflection loss of −50.16 dB, a broad effective absorption bandwidth of 7.44 GHz, and an ultrawide tunable range of 5.6 to 16.4 GHz. Benefiting from its exceptional mechanical properties and pressure-dependent tunability, the aerogel establishes a clear correlation between compression-induced capacitive response and MA performance. These features highlight its potential as a new generation of intelligent microwave absorbers with integrated sensing and adaptive regulation capabilities.

## Introduction

Currently, the deep integration of multiband and multimode communication systems is intensifying the complexity of electromagnetic radiation, leading to more intricate cross-interference that increasingly threatens both human health and the operational reliability of electronic devices [1–6]. Although considerable advances have been made through material-level innovation and structural design, the vast majority of current absorbers is static in nature, with the performance fixed during fabrication. Attempts at dynamic control, whether through engineered subwavelength structures or electrically or thermally responsive media, still suffer from fundamental trade-offs, including narrow adjustability, slow response, or high energy cost, leaving the challenge of practical broadband tunability unresolved [7–10]. It therefore remains a critical challenge to realize a simple, fully reversible tuning mechanism that is inherently integrated into a mechanically robust material system.

A pressure-tunable strategy based on the quarter-wavelength matching theory has emerged as a promising solution [11,12]. By adjusting the compression ratio, one can rapidly and reversibly modulate the match thickness, pore structure, and electromagnetic parameters, achieving stable and broadband regulation. Among the representative material systems explored for this purpose, carbon foams and graphite networks have attracted considerable attention due to their high dielectric tunability under compression. Nevertheless, their intrinsic load-bearing capacity remains limited. This is attributed to their porous and weakly cross-linked framework, weak interlayer interactions from sp^2^ carbon bonding, and inherent brittle fracture. Consequently, they are prone to structural failure under external stress [13]. Excessive contact between conductive skeletons under high compression can also lead to the formation of dense conductive networks, resulting in impedance mismatch and reduced absorption [7].

Polymer aerogels, owing to their exceptional elasticity, ultralow density, high specific surface area, and outstanding physicochemical stability, are regarded as the most promising candidates to overcome the above challenges [14,15]. However, conventional polymer aerogels (such as polyvinyl alcohol, polyimide [PI], and polyurethane) possess low intrinsic conductivity, lack polar groups and free carriers, and exhibit dielectric and magnetic parameters close to those of vacuum, which hinders effective microwave absorption (MA). To address this, researchers typically incorporate electromagnetic fillers (such as carbon nanotubes, graphene, and metal nanoparticles) to impart superior MA through multiple loss mechanisms, including electromagnetic scattering, coupling, and polarization losses [16–18]. Prior studies have shown that optimal MA depends on the homogeneous dispersion and effective isolation of fillers within the polymer matrix [19]. Nevertheless, achieving these requirements in practice faces multiple challenges: (a) Polymer aerogels possess weak mechanical strength due to high porosity and flexible skeletons, making them prone to brittleness and collapse; (b) interconnected pores generate capillary forces during drying, causing substantial volume shrinkage and reduced structural stability; (c) the lack of confinement in the polymer matrix results in filler aggregation and dielectric/magnetic inhomogeneity, thereby impairing MA. Although existing strategies (such as surface functionalization, plasma treatment, and covalent cross-linking) have partially addressed these issues, they often involve complex processing, cumbersome operations, and high costs, which greatly limit their practical application [20–22].

Here, we propose a strategy to construct a nested microaerogel structure that simultaneously enhances the mechanical strength and MA performance of PI aerogel. This microscopic network not only serves as a spatial scaffold to markedly improve mechanical robustness but also ensures high filler content with uniform dispersion, thereby imparting excellent MA performance. The resulting composite aerogel exhibits a compressive strength of 1.3 MPa, nearly 20 times that of pristine PI aerogels, and an effective absorption bandwidth (EAB) of 7.44 GHz covering the 5.6 to 16.4 GHz range. In addition, it combines other desirable features for microwave absorbers, including low density, impact resistance, and thermal insulation. Benefiting from its outstanding mechanical properties and pressure-dependent tunability, a coupling is further established between compression-induced capacitive response and MA performance, enabling precise detection and feedback regulation of electromagnetic characteristics via simple electrical signals. These unique functionalities exhibit broad application potential in areas such as intelligent electromagnetic regulation, adaptive MA systems, and integrated structure–function sensing, thereby offering new possibilities for expanding the practical application scenarios of MA materials.

## Results and Discussion

### Characterization on structure and morphology

**Fig. 1.**
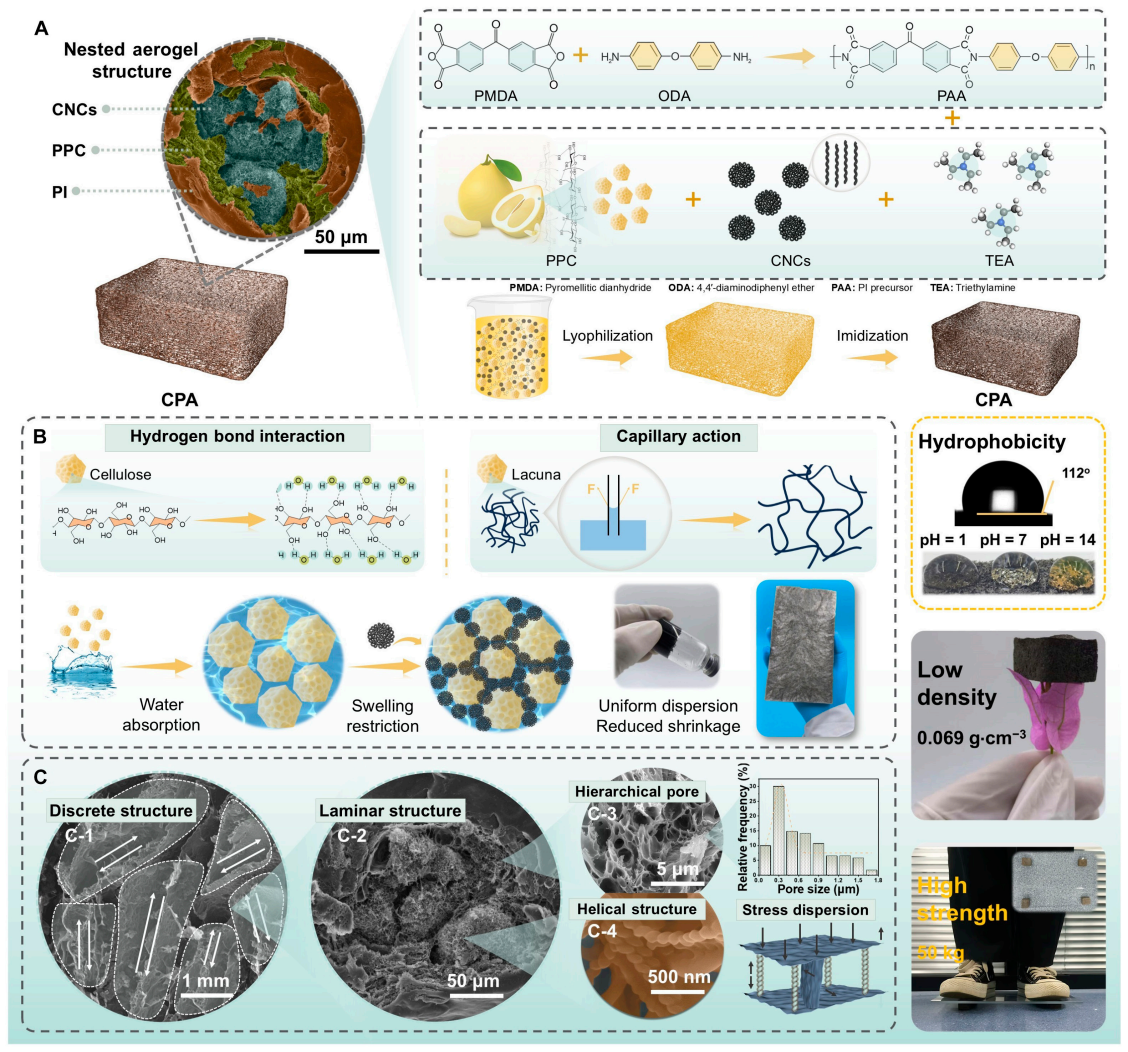
Fabrication process, microstructure, and multifunctional properties. (A) A schematic illustration of the fabrication process and digital images demonstrating its high strength, low density, and hydrophobic nature. (B) Schematic diagram of the formation mechanism of CPA. (C) SEM images, pore size distribution diagram, and microstructure schematic diagram of CPA.

**Fig. 2.**
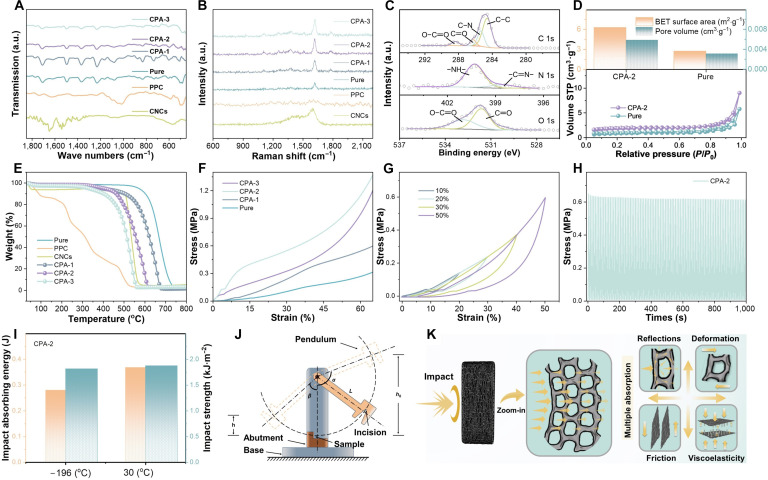
Characterization of structural and mechanical properties. (A) FTIR spectra. (B) Raman spectra. (C) High-resolution XPS profiles. (D) BET, specific surface areas, and total pore volumes at *P*/*P*_0_ = 0.95. (E) TGA curves. (F) Stress–strain curves. (G) Stress–strain curves under different compressive ratios. (H) Cyclic compression test at a strain of 50%. (I) Impact resistance performance test. (J) Schematic of impact pendulum and (K) shock wave absorption mechanism.

**Fig. 3.**
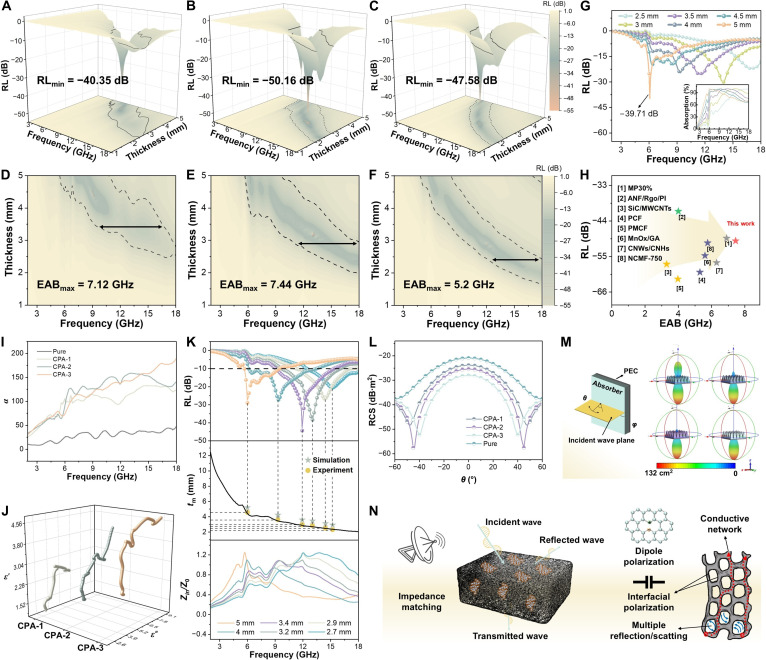
MA properties. (A to C) 3-dimensional (3D) and (D to F) 2D RL representations. (G) The RL values and corresponding absorption efficiencies. (H) Comparison of RL_min_ and EAB_max_ among previously reported absorbers. (I) The *α* and (J) Cole–Cole curves. (K) *λ*/4 matching model. (L) RCS simulated curves. (M) 3D radar wave scattering signals. (N) Scheme of microwave attenuation process.

**Fig. 4.**
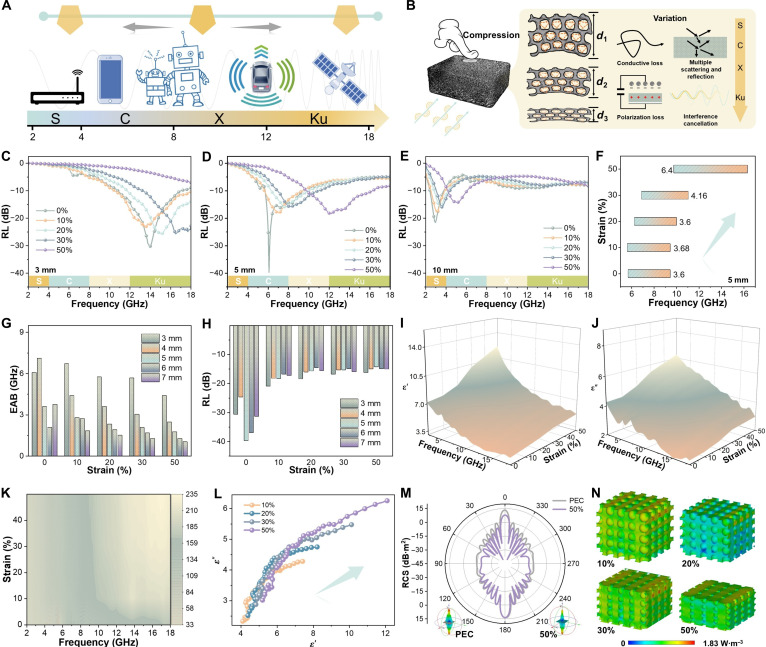
Tunable MA properties. Schematic diagram of (A) application scenarios and (B) tunable mechanism. (C to E) The RL values under various compression ratios. (F) The EAB traces values. (G) The EAB_max_ values and (H) RL_min_ values. (I) The *ε'*, (J) *ε"*, (K) *α*, and (L) Cole–Cole values. (M) The RCS curves and (N) 3D power loss density plots under different compression ratios.

Through the homogeneous mixing of natural pomelo-peel-derived cellulose micronetwork (PPC), helical carbon nanocoil microaerogels (CNCs), and an aqueous solution of PI precursors, followed by vacuum drying and subsequent imidization, a high-strength, hydrophobic PI/PPC@CNC composite aerogel (CPA) with a density of only 0.069 g·cm^−3^ can be fabricated (Fig. 1A). In this process, the PPC establish multiple hydrogen bonds with water molecules via the abundant hydroxyl (–OH) groups on their molecular chains (Fig. 1B) [23]. Meanwhile, the cellulose microtubes in PPC absorb water and swell via capillary action, thereby uniformly confining the CNCs within the PPC and ultimately constructing a gradient-nested PPC@CNC microaerogel structure (Figs. S1 to S3). The process details are outlined in the experimental procedures (Supplementary Materials). Figure 1C and Fig. S4 show scanning electron microscopy (SEM) images of CPA. The CPA exhibits a discrete, layered porous structure with a gradient distribution of pore sizes. This structural feature originates from the freezing step during the freeze-drying process of the PI precursor solution, where water crystallizes at low temperatures, causing the PI precursors and other components to precipitate between the forming ice layers. Subsequently, polymerization and cross-linking of PI molecules occur, during which the rigid PPC framework and the structural support provided by CNCs stabilize the pore morphology and substantially suppress volume shrinkage. It is worth noting that the irregular morphology of PPC and CNC powders introduces numerous ice nucleation sites into the system, promoting the formation of ice crystals in multiple directions and ultimately inducing the development of a discrete layered structure. The reduction in pore size and the increase in pore wall surface roughness correlate with the increasing CNC content (Fig. S5). As for PI aerogel, it suffers from severe shrinkage during the thermal annealing of its precursor, causing poor elasticity and high density, although it has a porous network with smooth cell walls (Fig. S6). Notably, PPC exhibits a porous structure (Fig. 1C-3 and Fig. S7), which provides excellent structural stability and offers spatial confinement for the formation of well-dispersed CNC networks, thereby supporting effective electromagnetic scattering and loss within the composite [24,25]. As shown in Fig. 1C-4 and Fig. S8, chiral helical CNCs are uniformly embedded within the PI layers, intertwining with the matrix to generate friction and mechanical interlocking, thereby markedly enhancing the mechanical strength. The chiral configuration further induces cross-polarization losses, leading to improved dielectric dissipation capability [6]. Meanwhile, the insulating PI layers suppress the formation of highly conductive networks during compression, effectively avoiding the impedance mismatch induced by such pathways [7]. In addition, the PI shell prevents the penetration of corrosive media such as acids and bases (water contact angle, 112°) and forms strong interfacial interactions with PPC through hydrogen bonding, thus improving the mechanical robustness of CPA. This structural design is inherently system independent; replacing the polymer matrix, the fibrous supporting architecture, or the functional fillers allows the concept to be broadly implemented across diverse material systems, underscoring its remarkable generalizability and scalability.

Fourier transform infrared (FTIR) spectra (Fig. 2A) reveal characteristic peaks of PI at 1,773 and 1,708 cm^−1^ (asymmetric and symmetric C═O stretching) and 1,362 cm^−1^ (C–N stretching), while PPC exhibits absorption bands at 1,050 to 1,150 cm^−1^ (C–O–C stretching) and 1,420 cm^−1^ (O–H bending) [26]. Raman spectra (Fig. 2B) show typical peaks of CNCs at ~1,380 cm^−1^ (D band) and 1,586 cm^−1^ (G band), and a distinct peak of PI at 1,618 cm^−1^ attributed to aromatic C═C stretching [27,28]. With increasing CNC content, the intensity of this peak is substantially enhanced, likely because of its overlap with the G band of CNCs, which further amplifies the Raman signal. X-ray photoelectron spectroscopy (XPS) analysis (Fig. 2C and Fig. S9) further demonstrates that the chemical composition of CPA is highly consistent with that of PI, PPC, and CNCs. All samples display prominent C 1s (~285 eV) and O 1s (~532 eV) peaks, while both PI and CPA exhibit an N 1s peak (~400 eV), thereby confirming the compositional features of the composite structure. The nitrogen adsorption–desorption isotherms (Brunauer–Emmett–Teller [BET]) reveal that both pure PI and CPA-2 exhibit typical type IV adsorption isotherms (Fig. 2D), indicating the coexistence of abundant micropores and mesopores within the aerogels. Further analysis of BET surface area and pore volume indicates that both the specific surface area and pore volume of CPA-2 are substantially higher than those of pure PI. These findings confirm that the incorporation of PPC and CNCs effectively enhances the porosity of CPA, which is crucial for achieving excellent impedance matching characteristics and enhancing microwave scattering and reflection [28]. Thermogravimetric analysis (TGA) curves (Fig. 2E and Fig. S10) indicate that pure PI aerogel exhibits the highest thermal stability, with major degradation occurring at ~550 to 700 °C. In contrast, PPC undergoes rapid weight loss starting at ~200 °C, showing the lowest stability, while CNCs display moderate stability with marked weight loss around 450 °C. The degradation temperature of CPA lies between those of PI and PPC, suggesting that the synergistic effect between PI and CNCs enhances the thermal stability of the composite.

The compressive stress–strain curves (Fig. 2F) show that all samples exhibit linear elastic deformation at the initial stage, followed by nonlinear deformation, indicating good toughness [29]. Pure PI aerogels display the lowest strength, reaching only ~0.35 MPa at 60% strain, whereas the strength of CPA is substantially enhanced, with CPA-2 achieving the highest value of ~1.15 MPa. In contrast, CPA-3 shows reduced strength due to excessive CNCs introducing interfacial defects that weaken the matrix. Cyclic compression curves (Fig. 2G and Fig. S11) further reveal that CPA-2 demonstrates stronger energy dissipation capability at higher strains; its cyclic fatigue test at 50% strain (Fig. 2H) shows that the stress stabilizes at ~0.64 MPa without noticeable degradation, which is attributed to the robust PI framework and the optimized synergistic interaction among components. These results confirm that CPA possesses excellent mechanical stability and fatigue resistance. Moreover, CPA-2 maintains stable impact absorption performance at both cryogenic temperature (–196 °C) and room temperature (30 °C) (Fig. 2I), reflecting structural robustness under extreme conditions. Its excellent impact resistance originates from the synergistic effect between the discrete layered porous structure and the high-strength PI matrix. During impact loading, the input energy is effectively dissipated through multiple mechanisms (Fig. 2J and K), including multiple reflections of stress waves within the heterogeneous structure, elastic–plastic deformation of the PI framework, interfacial friction between CNCs and the matrix, and intrinsic viscoelastic dissipation of the PI network [30,31]. The interplay of these mechanisms effectively alleviates impact loads and suppresses structural failure, endowing CPA with superior impact tolerance and making it a promising candidate for protective and load-bearing applications under extreme environments.

### MA performance and loss mechanisms

The MA performance of CPA was evaluated by transmission line theory (Eqs. S1 to S7) [32,33]. When reflection loss (RL) is below −10 dB, it is considered to indicate good MA capacity [34]. Compared with the pure PI aerogel, the CPA exhibits markedly enhanced MA performance (Fig. 3A to F and Fig. S12). This enhancement arises from the helical chiral structure, high electrical conductivity, and abundant polarization centers of the CNCs, which enable multiple loss mechanisms to synergistically strengthen the electromagnetic attenuation at an appropriate loading [35]. Among them, CPA-1 with low CNC content shows poor performance, with a minimum RL (RL_min_) of −40.35 dB and a maximum EAB (EAB_max_) of 7.12 GHz at a thickness of 3.70 mm. With moderate CNC content, CPA-2 exhibits excellent MA at 12.96 GHz, with an RL_min_ of −50.16 dB and an EAB_max_ of 7.44 GHz (6.32 to 13.76 GHz) at 3.90 mm. With higher CNC content, the MA performance decreases, with RL_min_ and EAB_max_ of −47.58 dB and 5.2 GHz, respectively. Figure 3G shows the RL values and corresponding absorption efficiencies of CPA-2 at different thicknesses. This MA performance surpasses that of recently reported absorbers (Fig. 3H and Fig. S13). The interaction between microwaves and the material is fundamentally determined by its intrinsic electromagnetic parameters [36]. Therefore, analysis of these parameters provides essential insight into the attenuation mechanism of CPA. During microwave propagation, the real part (*ε'*) and the imaginary part (*ε"*) of the complex permittivity represent the ability to store electric energy and dissipate it through dielectric loss, respectively [37–39]. Because of the weak polarization capability of PI, its dielectric constant is the lowest. With the increasing content of high-permittivity CNCs, the dielectric constant of CPA increases substantially (Fig. S14). Meanwhile, the ratio of polarization loss to conduction loss (*ε_p_″/ε_c_*″) gradually decreases with increasing CNC content (Fig. S15), indicating that the contribution of conduction loss to the overall dielectric loss becomes progressively more dominant [40–42]. As the composite contains no magnetic components, magnetic loss can be neglected, with the real part (*μ'*) close to 1 and the imaginary part (*μ"*) near 0 of the complex permeability (Fig. S16). In addition, both the attenuation coefficient (*α*) and the dielectric loss tangent (tan *δ_ε_*) of the CPA increase with the CNC content (Fig. 3I and Fig. S17), further indicating enhanced dielectric loss capability. As shown in Fig. 3J and Fig. S18, the Cole–Cole plot reveals the underlying dielectric loss mechanisms, where each depressed semicircle corresponds to a distinct polarization relaxation process. All the CPA display elongated tails and multiple semicircles in Cole–Cole plots, suggesting the coexistence of conduction loss and multiple polarization relaxation losses. Impedance matching is crucial for achieving superior MA, as it determines whether incident waves can effectively couple into the absorber and dissipate. When the impedance matching ratio (*Z* = |*Z_in_*/*Z_0_*|) approaches 1, microwaves can efficiently enter the absorber [43]. Figure 3K and Fig. S19 show the impedance matching behavior of CPA and their corresponding quarter-wavelength (*λ*/4) matching model.

Among them, CPA-2 shows the most favorable impedance matching, enabling optimal MA performance even with a moderate *α* value. Moreover, the absorption peak of the CPA shifts toward lower frequencies with increasing thickness. The experimental results agree well with simulations, confirming the role of interference in energy dissipation. The radar cross-section (RCS) is a key indicator for evaluating the far-field MA performance. To further validate the MA performance of CPA, Fig. 3L and M presents RCS simulations for a perfectly electric conductor (PEC) plate and a PEC plate coated with conical CPA. The radiation lobe structure and color distribution of the PI aerogel indicate the strongest reflection across the detection range [44], while the CPA demonstrates excellent reflection suppression. In summary, the CPA achieves outstanding MA performance through multiple polarization losses, conduction loss mechanisms, and internal porous architecture (Fig. 3N and Fig. S20) that promotes microwave reflection and scattering attenuation, demonstrating great promise for applications in electromagnetic protection.

### Tunable MA performance and loss mechanisms

Real-time tunable MA enabled by compressive strain endows next-generation absorbers with substantial advantages. This strain-responsive behavior allows real-time modulation of MA properties, facilitating adaptive applications in dynamically changing electromagnetic environments, including smart wearable electronics, automotive radar, reconfigurable stealth systems, and aerospace platforms (Fig. 4A). Compared with conventional absorbers possessing fixed structures, CPA can flexibly adjust its structure and electromagnetic properties by tuning the compression ratio, offering broad prospects for the development of intelligent, flexible, and miniaturized electromagnetic protection systems (Fig. 4B). Figure 4 systematically illustrates the tunable MA performance of CPA under varying compressive ratios. Within a strain range of 0 to 50%, the MA performance of CPA exhibits pronounced modulation behavior (Fig. 4C to E and Fig. S21 to S23). With increasing compression ratios, the absorption peak frequencies for all initial thicknesses shift toward higher frequencies: At an initial thickness of 3 mm (Fig. 4C), the peak absorption frequency rises from 10.5 to 18 GHz; at a thickness of 5 mm (Fig. 4D), it increases from 5.6 to 16.4 GHz; and at a thickness of 10 mm (Fig. 4E), the frequency shifts from 2.4 to 6.1 GHz.

Notably, for the CPA with an initial thickness of 5 mm, tuning the compression ratio effectively expands the absorption bandwidth (RL ≤ −10 dB) from the original C band to the Ku band (Fig. 4F). This coverage accounts for approximately 60% of the total measured frequency range, substantially exceeding the reported tunable absorption bandwidths of existing metamaterial absorbers. The EAB and RL of CPA with various initial thicknesses under different strain configurations were systematically analyzed (Fig. 4G and H). Remarkably, even at an initial thickness of only 3 mm, CPA maintains an RL below −30.6 dB with an EAB greater than 6.7 GHz. To date, microwave absorbers integrating frequency tunability, ultralow RL, and broadband performance at compact scales have been seldom reported.

The variation in electromagnetic response was also evident in the permittivity of CPA (Fig. 4I and J and Fig. S24 and S25), confirming that the electromagnetic properties of CPA were sensitive to applied compressive strain. As the compression ratio increased, a rise in *α* was observed, which played a role in modulating the attenuation behavior of the CPA (Fig. 4K). The Cole–Cole plots (Fig. 4L) revealed that the evolution of multiple semicircular arcs and changes in the linear tail region revealed dynamically adjustable polarization relaxation and conductive loss behaviors, attributable to structural rearrangement of the conductive network and pore architecture during compression process [45]. Furthermore, compressive strain induces variations in impedance matching (Figs. S26 and S27). When the compression becomes excessive, the deterioration of MA performance is likely attributable to excessive pore collapse and conductive network overpercolation [7,11]. To further verify the dynamic tunability, we obtained the simulated RCS profiles and energy dissipation maps for PEC surfaces with and without CPA coating (Fig. 4M and N and Figs. S28 and S29). The distinct changes in lobe patterns and energy intensity confirmed that CPA dynamically modulate surface reflection. Notably, the RCS value at a 69° incident angle decreased from −29.7 to −43.5 dB·m^2^, and the reduction extended over nearly the full angular range. Compression-induced variations in energy dissipation density further supported the strain-responsive absorption capability. Thus, the results highlighted the promising potential of CPA as dynamically tunable microwave absorbers for applications in stealth technologies and electromagnetic pollution mitigation.

### Application of multifunction

**Fig. 5.**
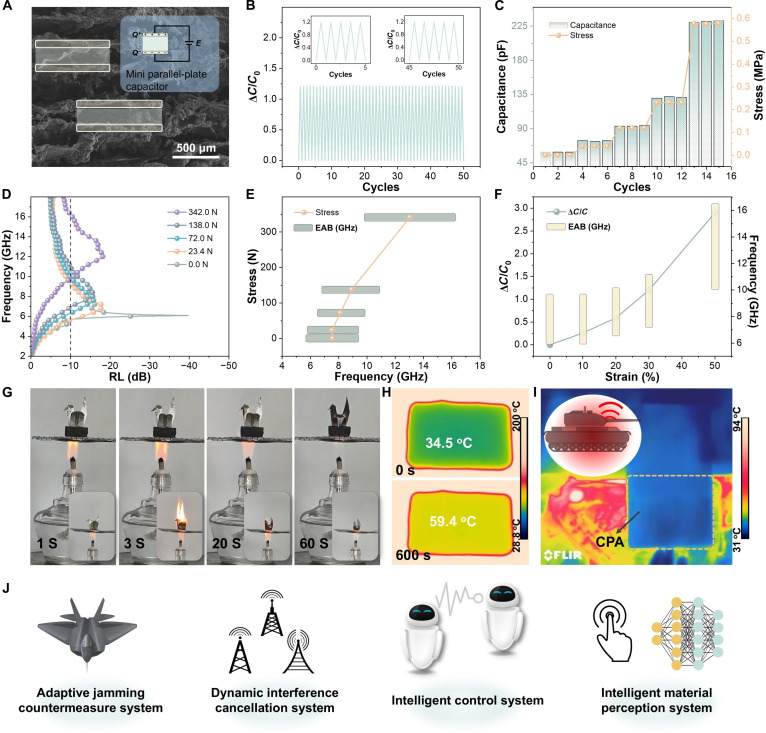
Multifunctional application scenarios. (A) SEM image. (B) Sensing performance. (C) Capacitance values. (D) RL curves and (E) EAB traces under varying stress levels. (F) EAB traces corresponding to change in capacitance values. (G) Digital photographs of CPA-2 burning under an alcohol lamp. Infrared thermal images of CPA-2 placed (H) on a heating stage (200 °C) and (I) on a car engine. (J) Application scenarios of CPA.

Tunable microwave absorbers are highly desired in typical applications such as radar stealth, intelligent communication, and dynamic interference suppression. In these scenarios, absorbers are expected to respond rapidly to structural deformation or external perturbations, dynamically adjusting their absorption behavior to achieve real-time microwave attenuation and effective control. These scenarios place more stringent requirements on absorbers: Beyond stable and tunable MA performance, they should be capable of producing identifiable physical signals during structural transitions to enable system-level real-time feedback and closed-loop regulation [46,47]. Against this background, this study proposed and experimentally validated a capacitance-based tuning strategy for MA, which overcame the limitations of conventional approaches relying on external electromagnetic measurements. As shown in Fig. 5A, the layered structure of the CPA undergoes gradient deformation under compression, where each layer experiences a different degree of stress, thereby enhancing the sensitivity of the overall structure to subtle pressure changes. In addition, the tiny gaps between layers lead to effective changes in the electrode distance (*d*) during compression, further amplifying the capacitive response (Fig. S30). As illustrated in Fig. 5B and C and Figs. S31 and S32, the CPA exhibited pronounced and stable capacitance variations at different compression ratios. Precise correlations can be observed between stress and RL, stress and EAB, relative capacitance variation and EAB, as well as applied pressure and absorption efficiency (Fig. 5D to F and Fig. S33). The resulting “electrical–electromagnetic” coupling mechanism established a quantifiable feedback pathway from mechanical strain to absorption behavior. It opens a new avenue for designing intelligent tunable absorbers with integrated capabilities for pressure adaptation and feedback. This concept represents a critical shift in the design paradigm of MA materials from passive-responsive tuning to actively perceptive regulation.

To ensure long-term operational reliability in real-world environments, MA materials must withstand complex and often extreme service conditions, including localized heating induced by electromagnetic radiation, exposure to external heat sources, and even direct flame contact [48–50]. Therefore, imparting excellent flame retardancy and thermal insulation capability is essential to ensure structural integrity and operational stability in high-temperature service environments. In flame resistance tests (Fig. 5G), the CPA was directly exposed to the flame of an alcohol lamp for 60 s. Remarkably, the sample maintained its structural integrity without any signs of melting or continuous combustion, demonstrating outstanding thermal stability and fire resistance. This performance originates from the synergistic effect between the thermally stable PI matrix and the porous structure, which effectively suppresses flame propagation. Thermal insulation tests (Fig. 5H) show that, after continuous heating at 200 °C for 600 s, the surface temperature of CPA increases only from 34.5 to 59.4 °C, demonstrating excellent thermal insulation capability. Infrared imaging experiments (Fig. 5I) further reveal that the surface temperature of regions covered with CPA drops from 94 to ~31 °C, forming a low-emissivity zone that effectively conceals thermal signals and thus endows the material with effective infrared stealth performance. Benefiting from its microwave tunability, thermal stability, infrared shielding capability, and mechanical adaptability, CPA can serve as a multifunctional electromagnetic platform (Fig. 5J). It is suitable for applications such as adaptive interference suppression, thermal–electromagnetic protection, and intelligent sensing interfaces, thereby providing a foundation for the development of next-generation integrated and intelligent electromagnetic devices.

## Conclusion

In summary, we have developed a PI composite aerogel with a gradient-nested microaerogel architecture. This design effectively overcomes the poor dispersibility of high-content fillers and the structural collapse of conventional aerogels, achieving a nearly 20-fold enhancement in mechanical strength while maintaining excellent MA performance. The aerogel exhibits an EAB of 7.44 GHz and an RL_min_ of −50.16 dB, and its frequency response can be tuned within 5.6 to 12.4 GHz by simply adjusting the compression ratio. Moreover, the aerogel possesses multiple desirable characteristics for microwave absorbers, including low density, impact resistance, and thermal insulation. Notably, on the basis of the correlation between capacitive response and MA during pressure tuning, electromagnetic properties can be precisely detected and modulated through simple electrical signals. Such an approach provides a feasible pathway for developing intelligent microwave absorbers with integrated sensing and adaptive modulation capabilities.

## Materials and Methods

Information about the materials and methods used in this work is available in the Supplementary Materials.

## Data Availability

No data were used for the research described in the article.

## Supplementary Materials

Supplementary 1 Experimental Section Figs. S1 to S33
