# Supplementary material for: Gradient-Nested Organic/Inorganic Aerogels Achieve High Mechanical Strength and Feedback-Tunable Microwave Absorption
Source: Research (Wash D C). 2026 Jan 14;9:1074. doi: 10.34133/research.1074 (PMC12799915; doi:10.34133/research.1074)
Supplement: Supplementary 1 — Experimental Section Figs. S1 to S33 [file research.1074.f1.docx]

Supporting Information

**Gradient-Nested Organic/Inorganic Aerogels Achieve High Mechanical Strength and Feedback-Tunable Microwave Absorption**

Xiao Liu^a,b^, Zhike Si^a,b^, Yongxin Qian^a,b^, Lihong Wu^a,b^, Xuefei Xu^a,b^, Gengping Wan^a,b,c^*, Guizhen Wang^a,b,c^*

^a^ Institute of Electromagnetic Protection Materials and Spectral Innovation Technology，State Key Laboratory of Tropic Ocean Engineering Materials and Materials Evaluation， School of Material Science and Engineering, Hainan University, Haikou, Hainan 570228, China.

^b^ Center for New Pharmaceutical Development and Testing of Haikou, Center for Advanced Studies in Precision Instruments, Haikou, Hainan 570228, China.

^c^ Key Laboratory of Pico Electron Microscopy of Hainan Province, Center for Advanced Studies in Precision Instruments, Haikou, Hainan 570228, China.

*Corresponding author. E-mail address: wangengping@hainanu.edu.cn (Gengping Wan); wangguizhen@hainanu.edu.cn (Guizhen Wang).

**Experimental Section**

**Materials**

Acetylene (99%) was purchased from Hainan Jiateng Chemical Gas Co. Ltd. (Hainan, China). Pomelo peel powder (PPC) was obtained from locally grown honey pomelos in Hainan Province, China. Pyromellitic dianhydride (PMDA, 99.8%), 4,4′-diaminodiphenyl ether (ODA, 98%), N,N-dimethylacetamide (DMAc, 99%), and Triethylamine (TEA, AR, 99%) were purchased from Macklin. All reagents were used as received, without further purification.

*Preparation of CNCs*

The CNCs were synthesized by chemical vapor deposition using acetylene as a carbon source and copper nanoparticles as catalysts at 250^o^C followed by a heat treatment at 900^o^C in an Ar atmosphere for 2 h as reported previously.

*Preparation of PPC*

Fresh pomelo peels were first washed with deionized water to remove surface impurities. The white spongy inner layer of the peel was carefully sliced using a clean stainless-steel blade. The obtained peel pieces were then subjected to freeze-drying (−50^o^C) for 48 h to completely remove moisture. After lyophilization, the dried pomelo peel was ground into fine powder using a high-speed mechanical grinder and subsequently sieved through a 100-mesh screen to ensure uniform particle size. The resulting pomelo peel powder (PPC) was collected and stored in a sealed container for further use.

*Preparation of PI precursor*

The PI precursor (PAA) was synthesized using a reported method. Typically, the homogenous PAA solution was obtained by dispersing ODA in DMAc followed by adding a certain amount of PMDA under vigorous mechanical stirring for 1 h. The resultant solution was then transferred into deionized water and the precipitate was washed and dried at low temperature to avoid the degradation of PAA. Aqueous solution of PAA was obtained by dissolving PAA of 0.05 g in water (1 mL) with 0.01 g TEA.

*Preparation of CPA*

The PPC (0.50 g) and CNCs (0.30 g) were added to the PAA solution (50 mL), followed by continuous stirring for 6 h. The resultant uniform mixture was frozen for 24 h, followed by lyophilization in a freeze-dryer for 72 h. The obtained PAA/CNCs aerogel was thermally annealed at 300^o^C in nitrogen atmosphere for 2 h to convert PAA to PI. The compositions of the aerogels were adjusted by altering the amount of CNCs added. The samples fabricated with different mass ratios of 1:10, 3:20, and 1:5 of CNCs to PAA and PPC were designated as CPA-1, CPA-2, and CPA-3, respectively.

**Characterization**

The morphology of the sample was analyzed by scanning electron microscopy (SEM, Thermo Scientific Verios G4 UC) and transmission electron microscopy (TEM, Talos F200X G2). The phase and crystal structure were determined by X-ray diffraction (XRD, Smart Lab II, Cu Kα radiation). Fourier transform infrared (FTIR) spectrum was characterized on a FTIR spectrometer (Bruker, TENSOR 27). X-ray photoelectron spectroscopy (XPS) data were recorded by AXIS SUPRA. Raman spectra were acquired using inVia Reflex (Renishaw) with a 514 nm laser. Thermogravimetric analyses (TGA) were conducted on TL9000. Cyclic compression performance was measured by an electronic universal testing machine (ETM series, WANCE). The impact resistance of the samples was evaluated using a pendulum impact test (−196°C via liquid-nitrogen immersion; 30°C under ambient conditions; PIT501J, WANCE). The contact angles of samples were measured using the DropMeterTM Experience A-300 (Ningbo Haishu Mai Time Testing Technology Co. LTD). The temperature of the sample was monitored with an infrared thermal imager (FLIR E6). The capacitive signals were measured by a digital multimeter (Tektronix, DMM6500). The electromagnetic parameters were evaluated in the frequency range of 2−18 GHz using a network analyzer (Ceyear, 3672B-S). The bulk aerogel samples were precisely machined into toroidal specimens (inner diameter 3.04 mm, outer diameter 7.00 mm, thickness 2.00 mm) compatible with the coaxial transmission line fixture and were subsequently secured with paraffin.

**Equations**

The reflection loss (RL) values were obtained by transmission line theory, which could be summarized as the following equations:

$Z_{in}=Z_{0}\sqrt{{\mu_{r}}/{\varepsilon_{r}}}\tanh[j({2\pi fd}/{c)\sqrt{\mu_{r}\varepsilon_{r}}}]$ (S1)

$RL=20\lg\left| {(Z_{in}-Z_{0})}/{(Z_{in}+Z_{0})} \right|$ (S2)

where *Z_in_* is the input impedance of the absorber, *Z_0_* is the impedance of free space, $\mu_{r}$ is the relative complex permeability ($\mu_{r}=\mu'-i\mu"$), $\varepsilon_{r}$ is the complex permittivity ($\varepsilon_{r}=\varepsilon'-i\varepsilon"$), *d* is the thickness of absorber, *c* and *f* are velocity and frequency of light, respectively.

In general, the dielectric loss can be described by Debye’s theory:

$\varepsilon'=\varepsilon_{\infty}+\frac{\varepsilon_{s}-\varepsilon_{\infty}}{1+\left( \omega\tau_{0} \right)^{2}}$ (S3)

$\varepsilon"=\frac{\omega\tau_{0}\left( \varepsilon_{s}-\varepsilon_{\infty} \right)}{1+\left( \omega\tau_{0} \right)^{2}}+\frac{\sigma}{\omega\varepsilon_{0}}$ (S4)

where, $\varepsilon_{\infty}$ is the relative dielectric permittivity in the high-frequency limit, *𝜏* is the polarization relaxation time, *𝜔* is the angular frequency, $\varepsilon_{s}$ is the static permittivity, $\varepsilon_{0}$ is the dielectric constant of vacuum, *σ* is the electrical conductivity.

The attenuation constant (*α*) represents the attenuation capacity of electromagnetic, which is usually expressed as follows:

$\alpha=\frac{\sqrt{2}\pi f}{c}\times\sqrt{\left( \mu"\varepsilon"-\mu'\varepsilon' \right)+\sqrt{\left( \mu"\varepsilon"-\mu'\varepsilon' \right)^{2}+\left( \mu"\varepsilon'+\mu'\varepsilon" \right)^{2}}}$ (S5)

For the relaxation losses, the Cole-Cole plots are used to analyze the polarization mechanism, according to the following equation:

$\left( \varepsilon'-\frac{\varepsilon_{s}+\varepsilon_{\infty}}{2} \right)^{2}+\left( \varepsilon^{''} \right)^{2}=\left( \frac{\varepsilon_{s}+\varepsilon_{\infty}}{2} \right)^{2}$ (S6)

The quarter-wavelength (*λ*/4) theory can be used to explain the relationship between matching thickness (*t_m_*) and absorption peak frequency (*f_m_*), which can be expressed as follows:

$t_{m}=\frac{n\lambda}{2}=\frac{nc}{4f_{m}\sqrt{\left| \varepsilon_{r}\mu_{r} \right|}} \left( n=1, 3,5\ldots. \right)$ (S7)

**Figures**


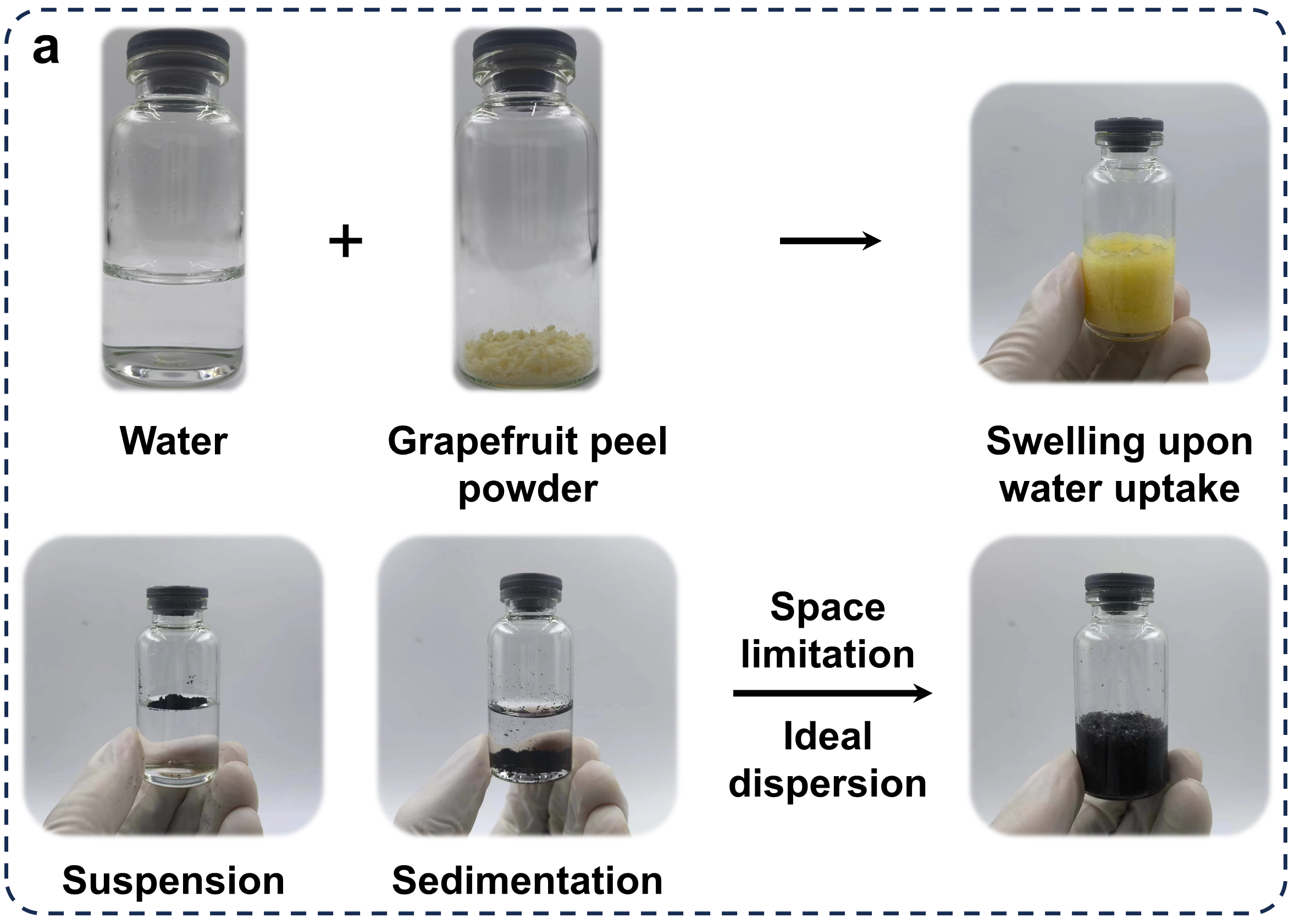


**Fig. S1.** Digital photographs of the water absorption swelling and uniform dispersion of CNCs in PPC.


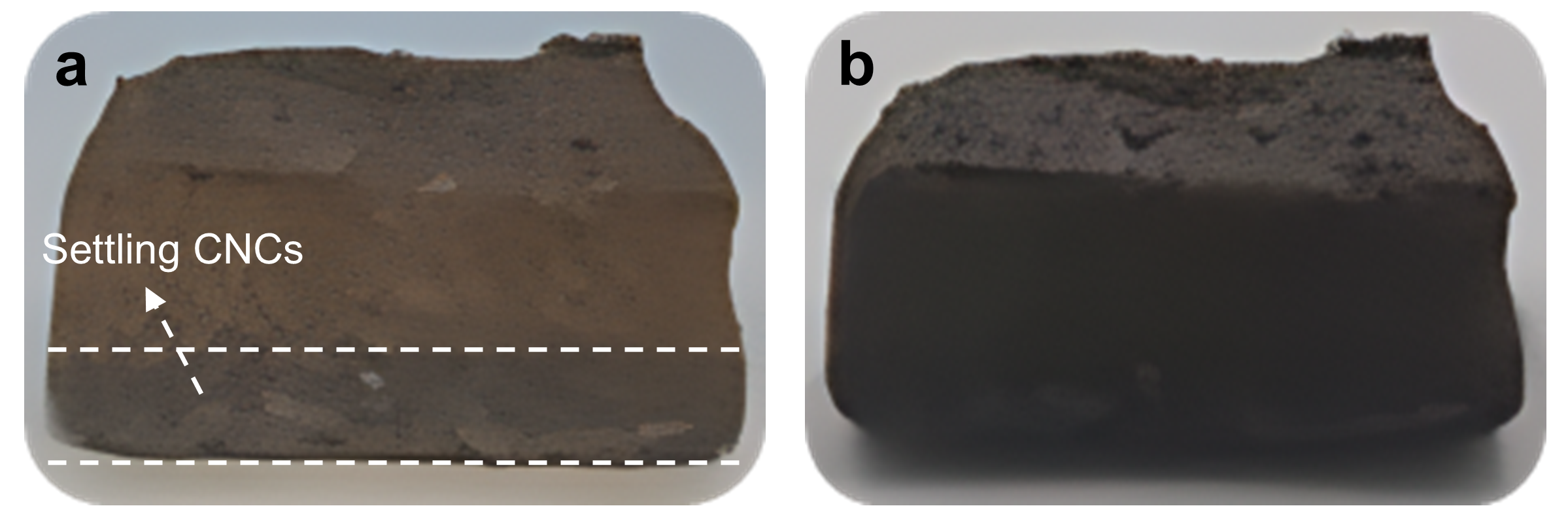


**Fig. S2.** Digital photographs of CPA in the (a) absence and (b) presence of PPC.


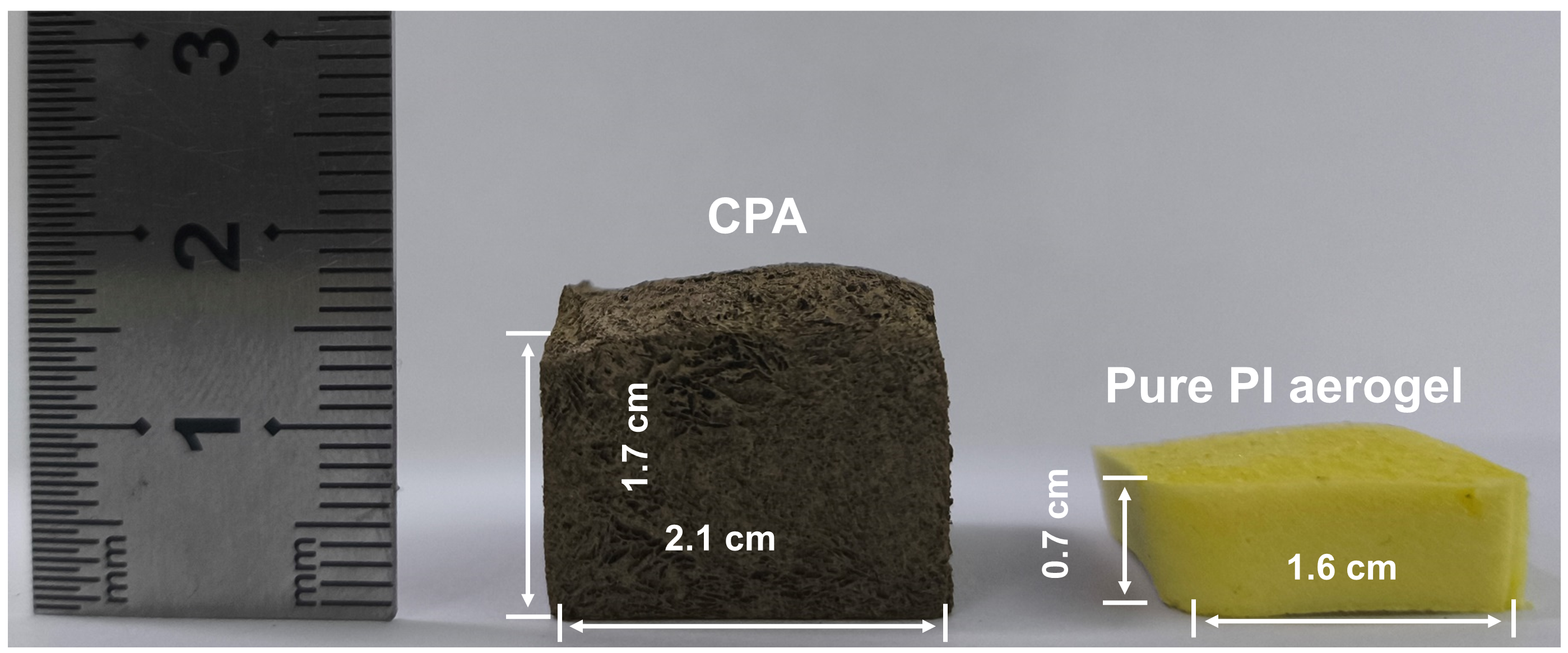


**Fig. S3.** Digital photographs of CPA and pure PI aerogel.


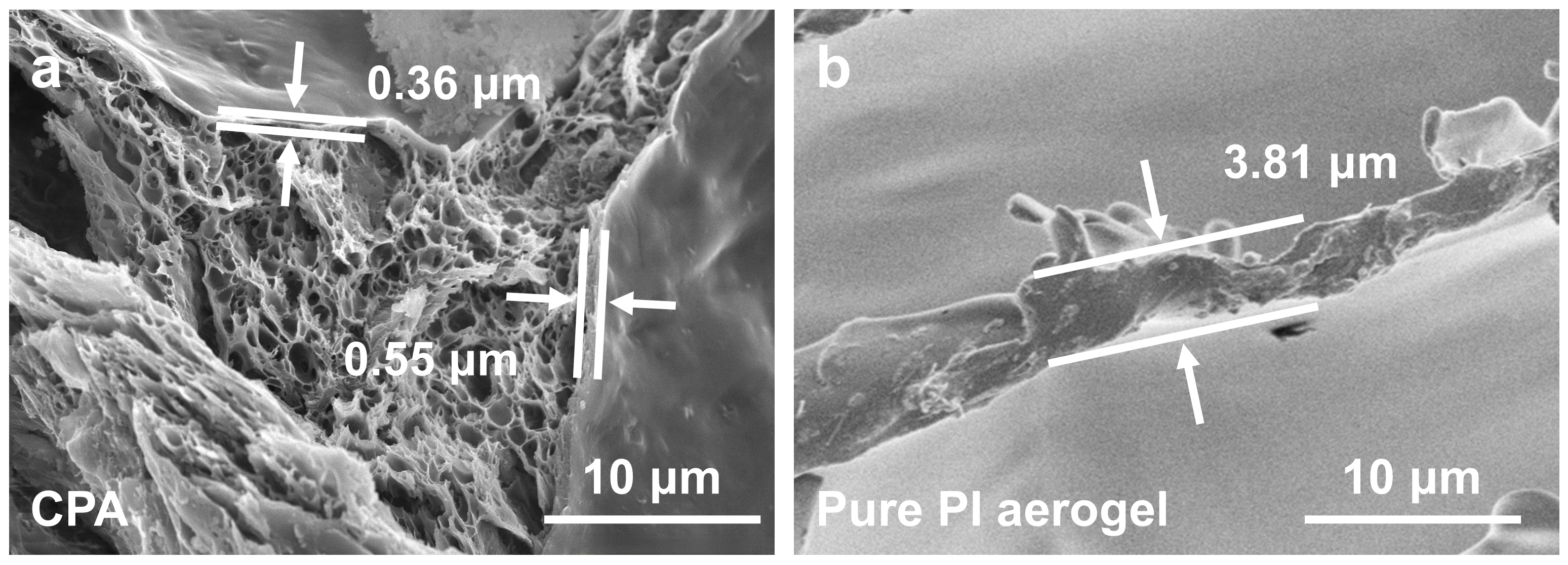


**Fig. S4.** SEM images of the PI layer thickness of CPA and pure PI aerogels.


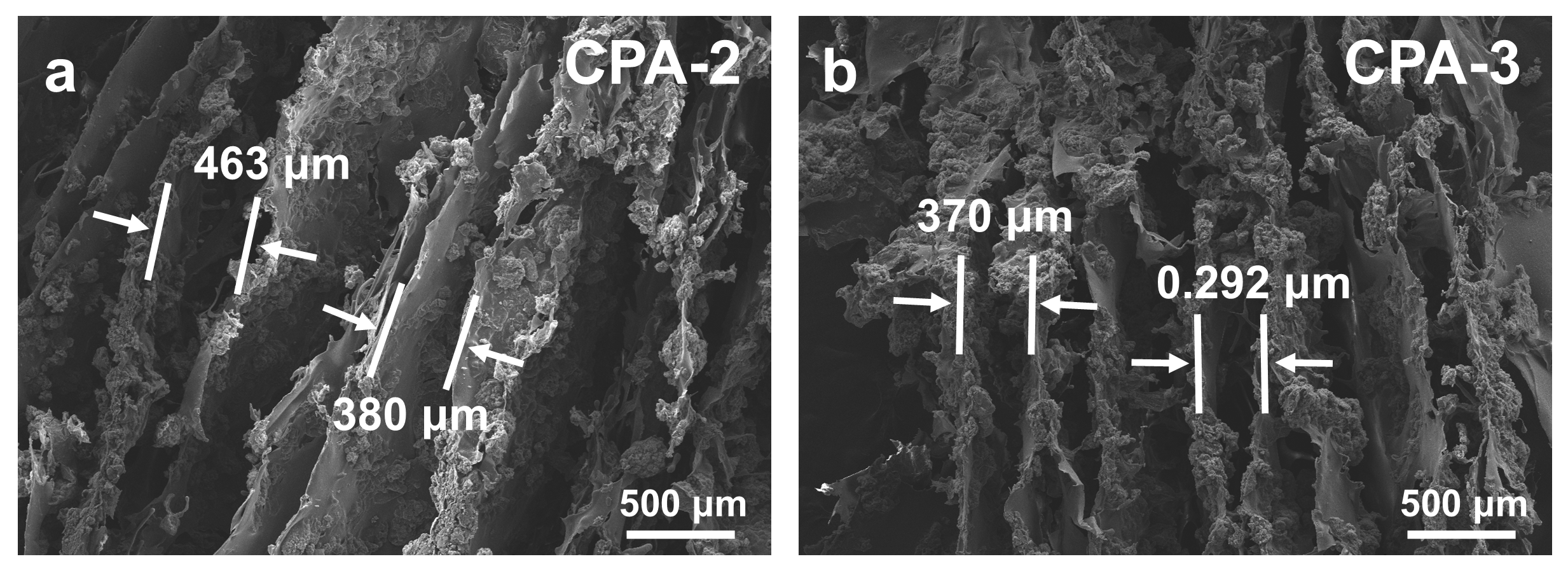


**Fig. S5.** SEM images of (a) CPA-2 and (b) CPA-3.


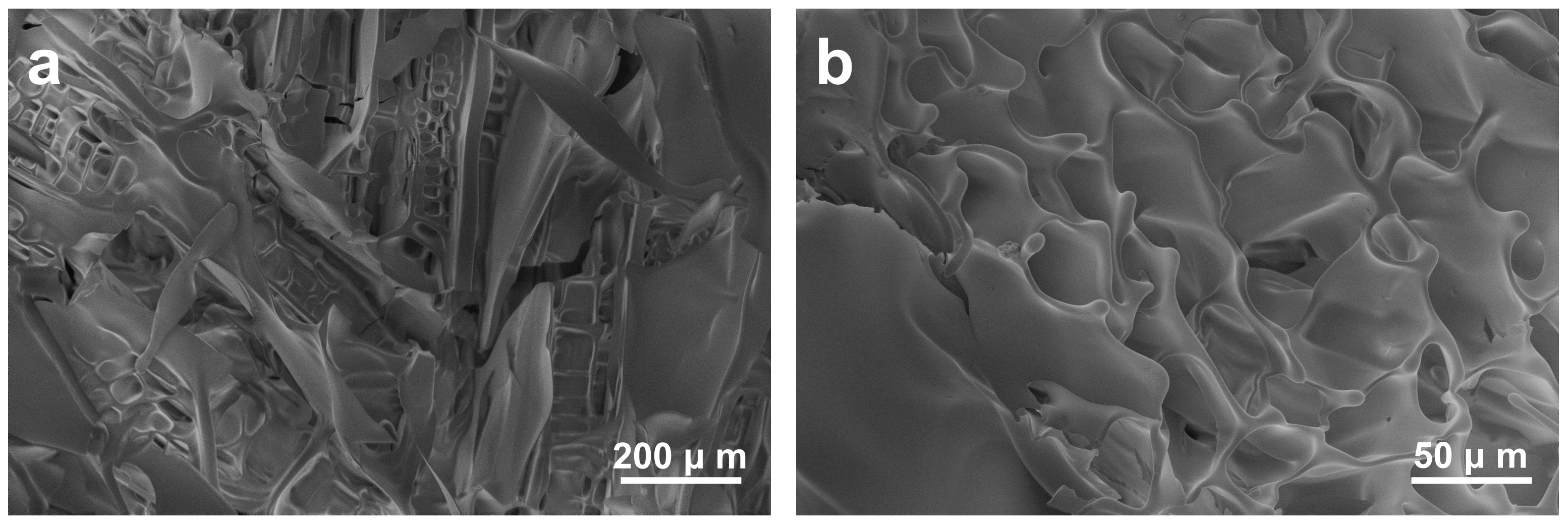
**Fig. S6.** (a, b) SEM images of pure PI aerogel.


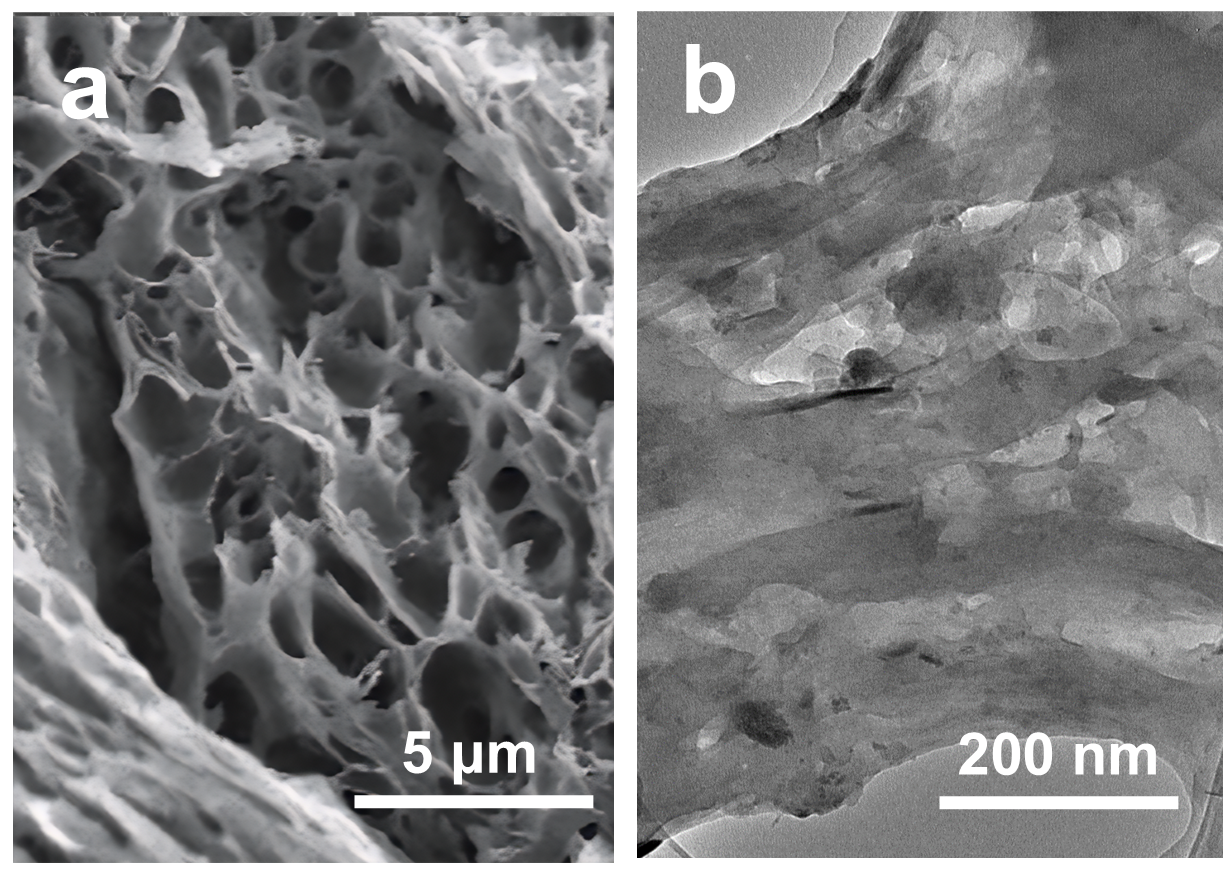


**Fig. S7.** (a) SEM and (b) TEM images of PPC.


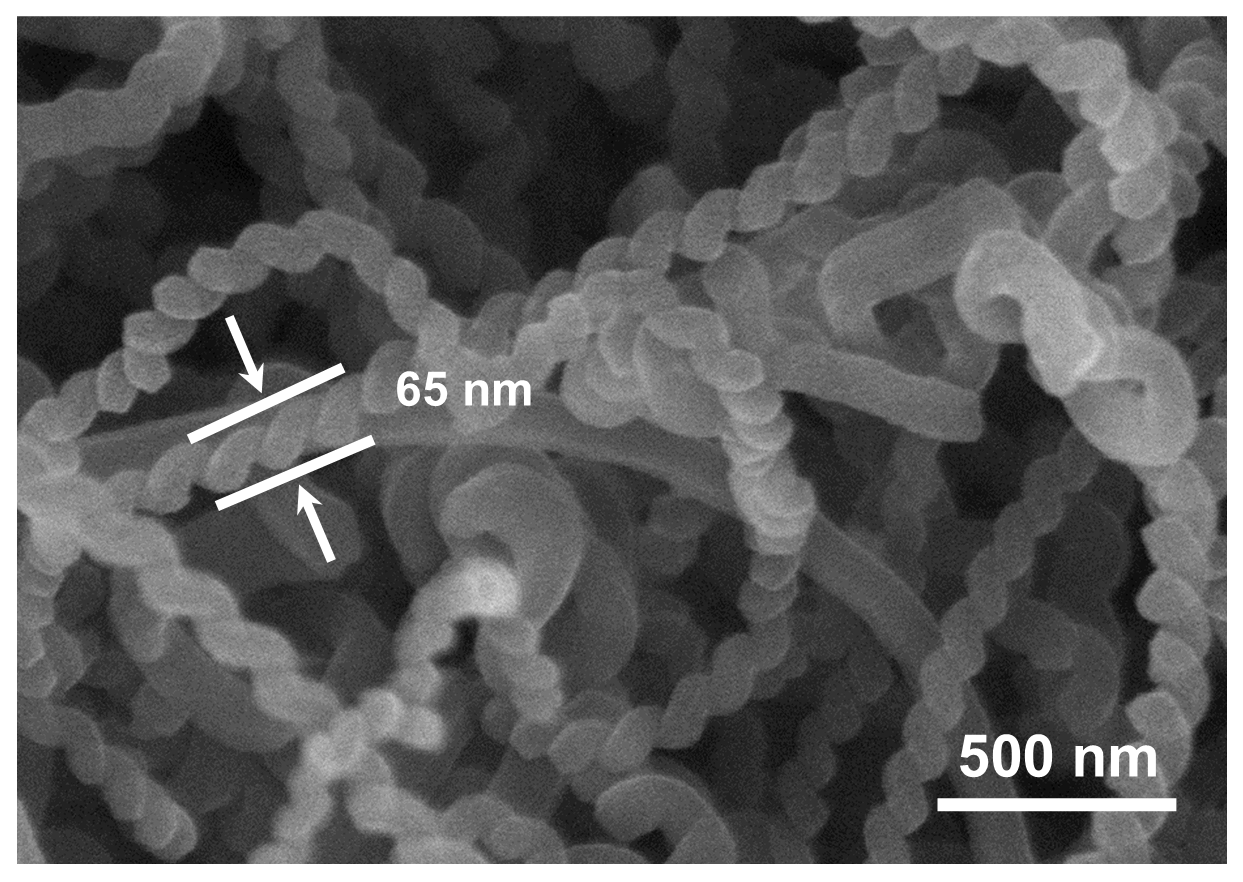


**Fig. S8.** SEM image of CNCs.


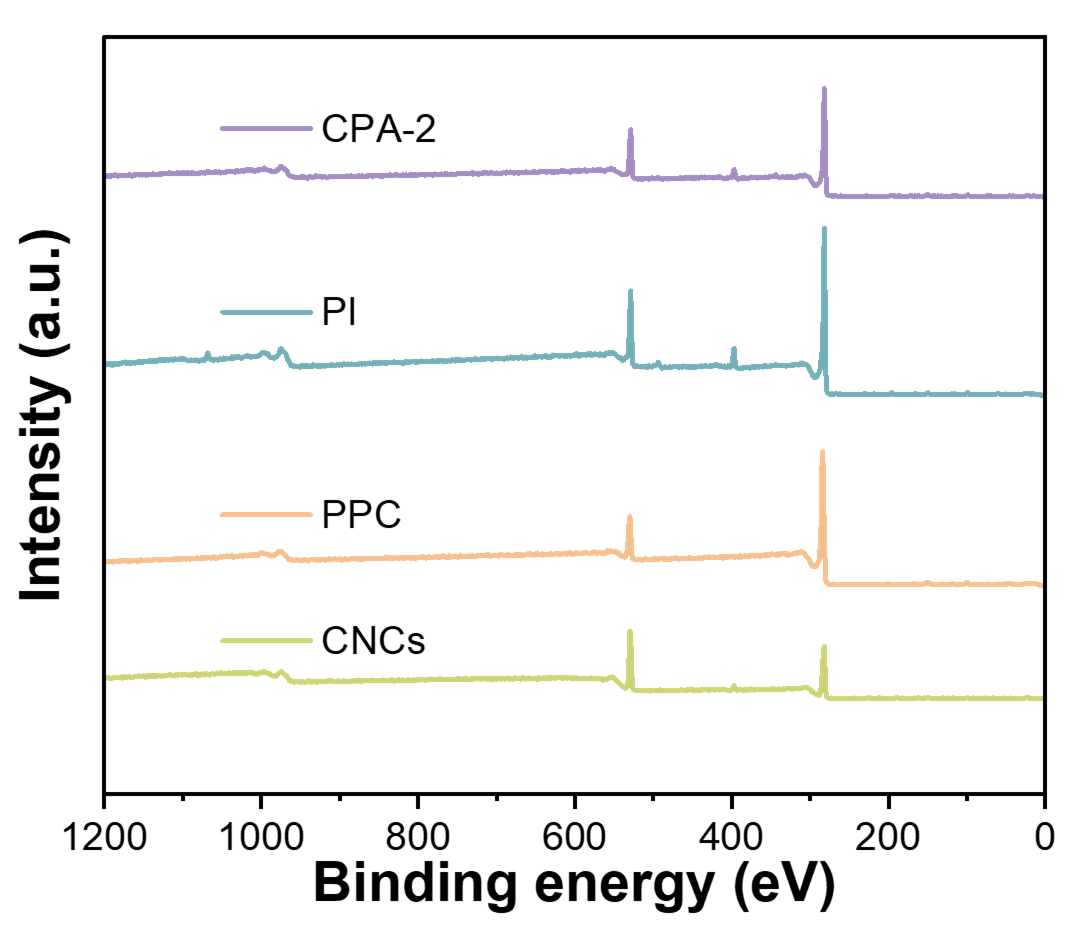


**Fig. S9.** XPS spectra of all samples.


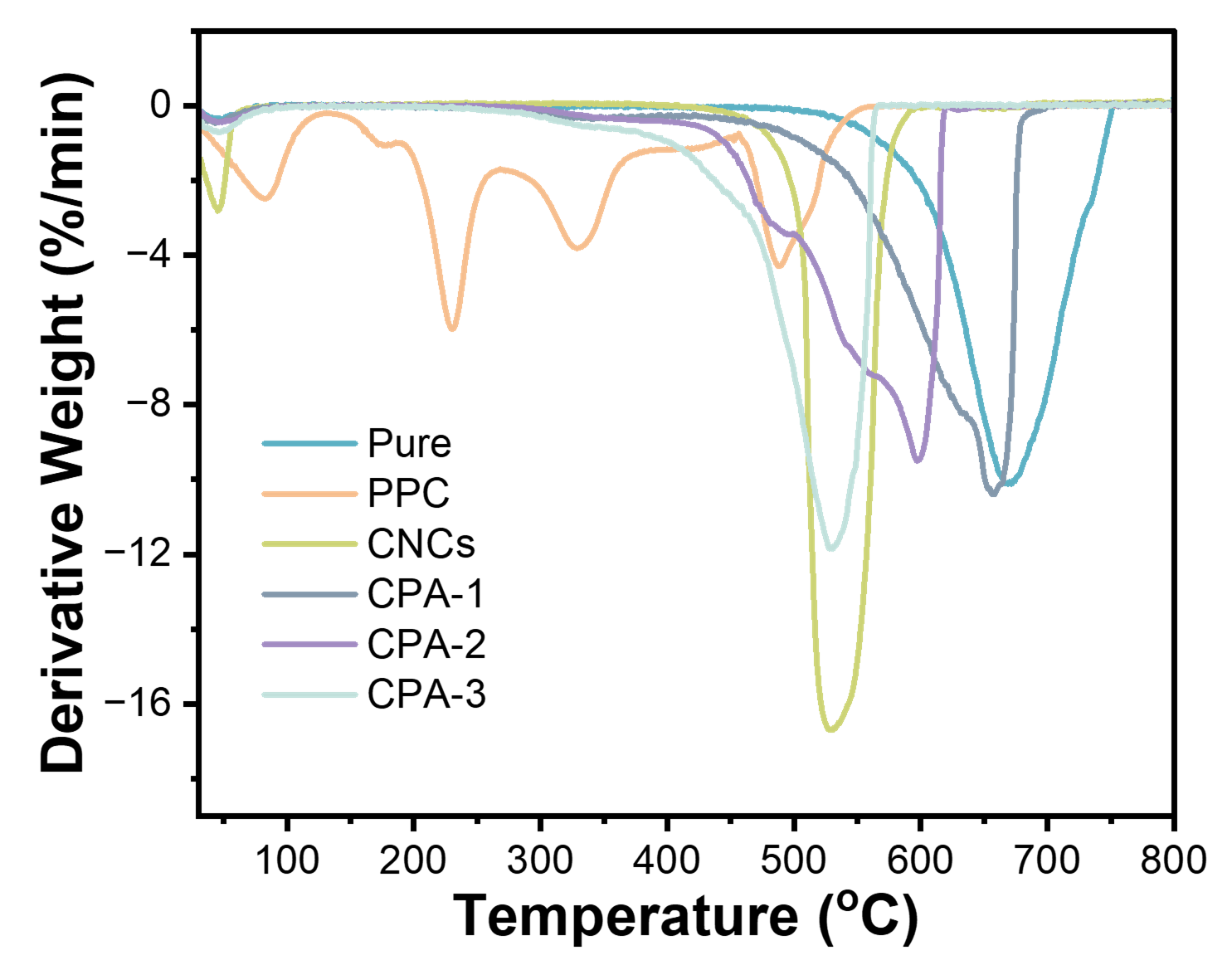


**Fig. S10.** DTG curves of all samples.


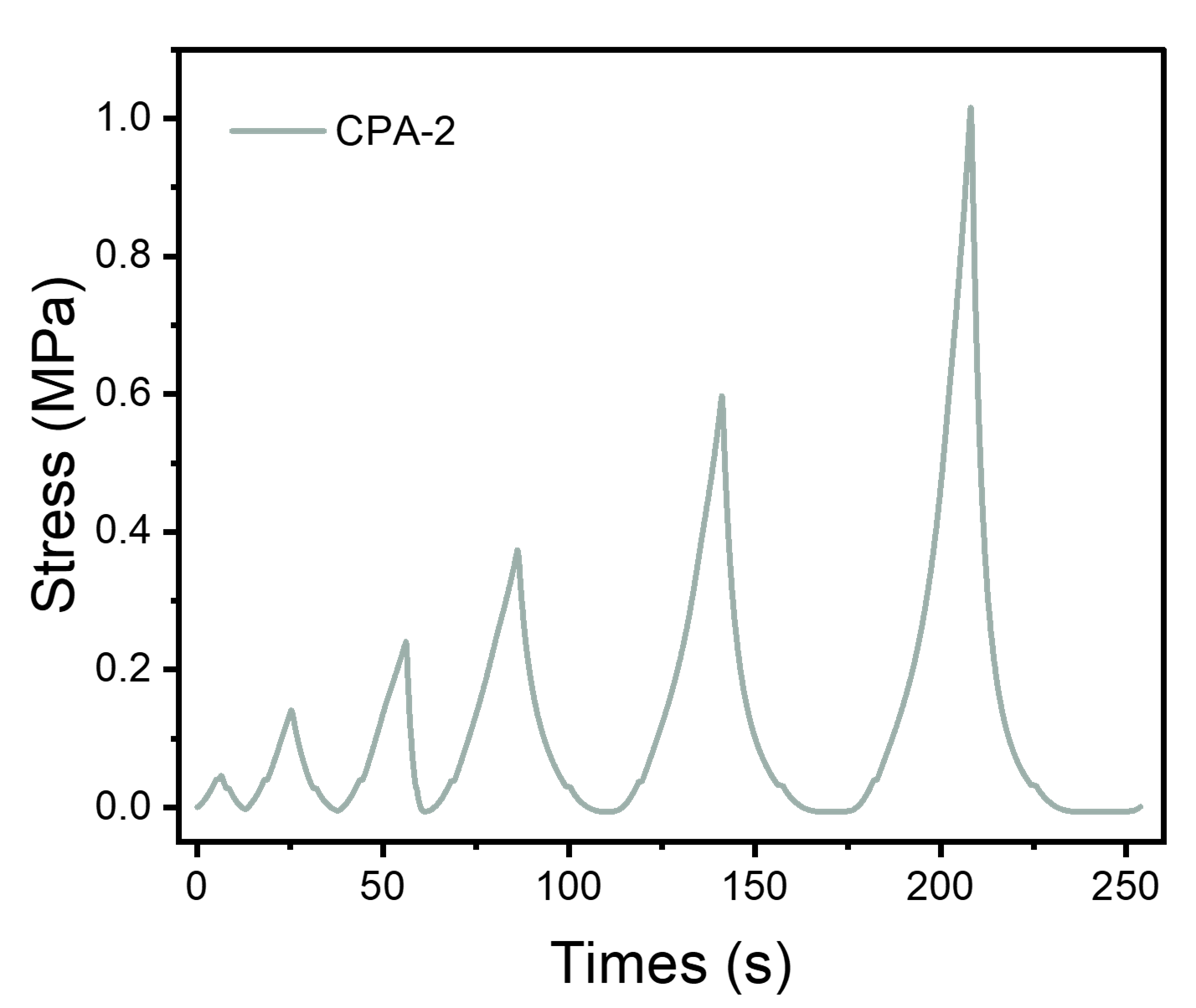


**Fig. S11.** The stress-time curve corresponding to different compression ratios.


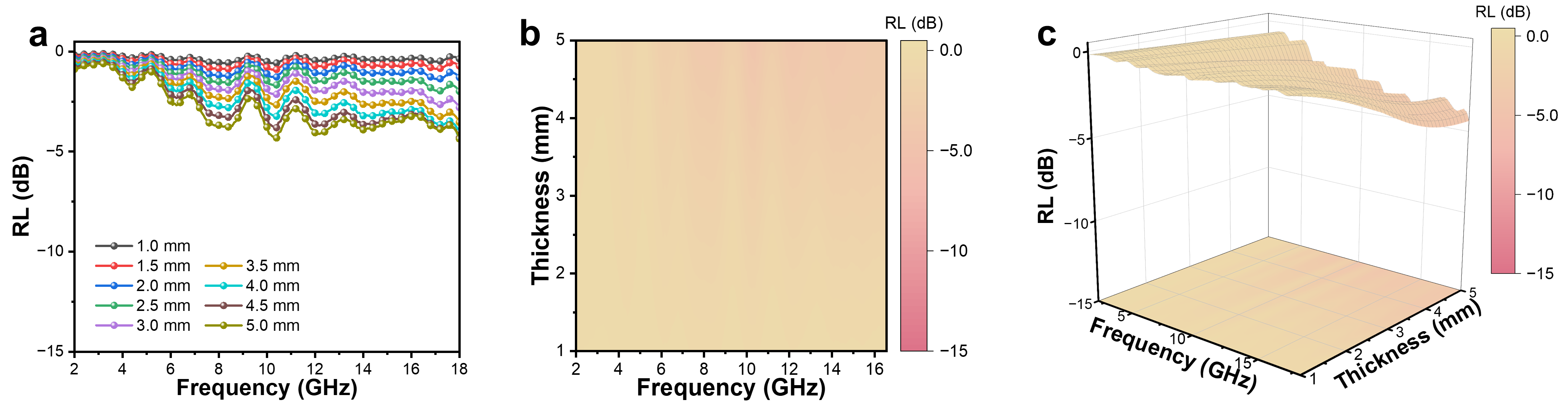


**Fig. S12.** The a) 1D, b) 2D, and c) 3D RL representations of pure PI aerogel.


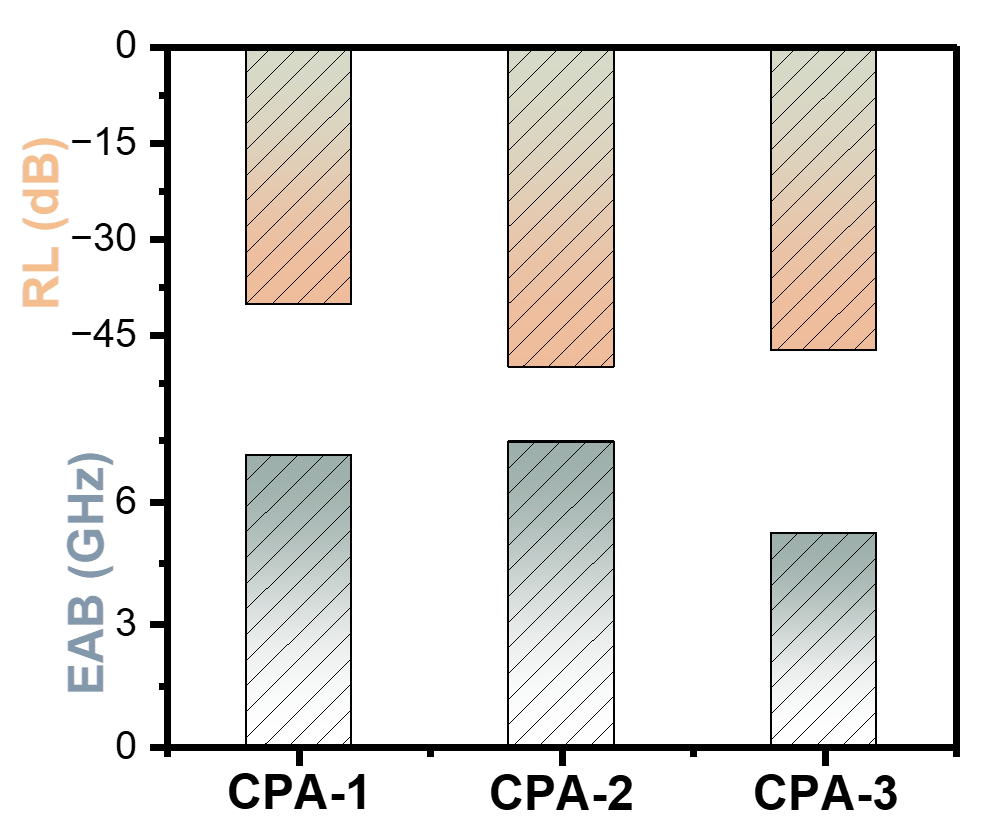


**Fig. S13.** The RL and EAB values of CPA.


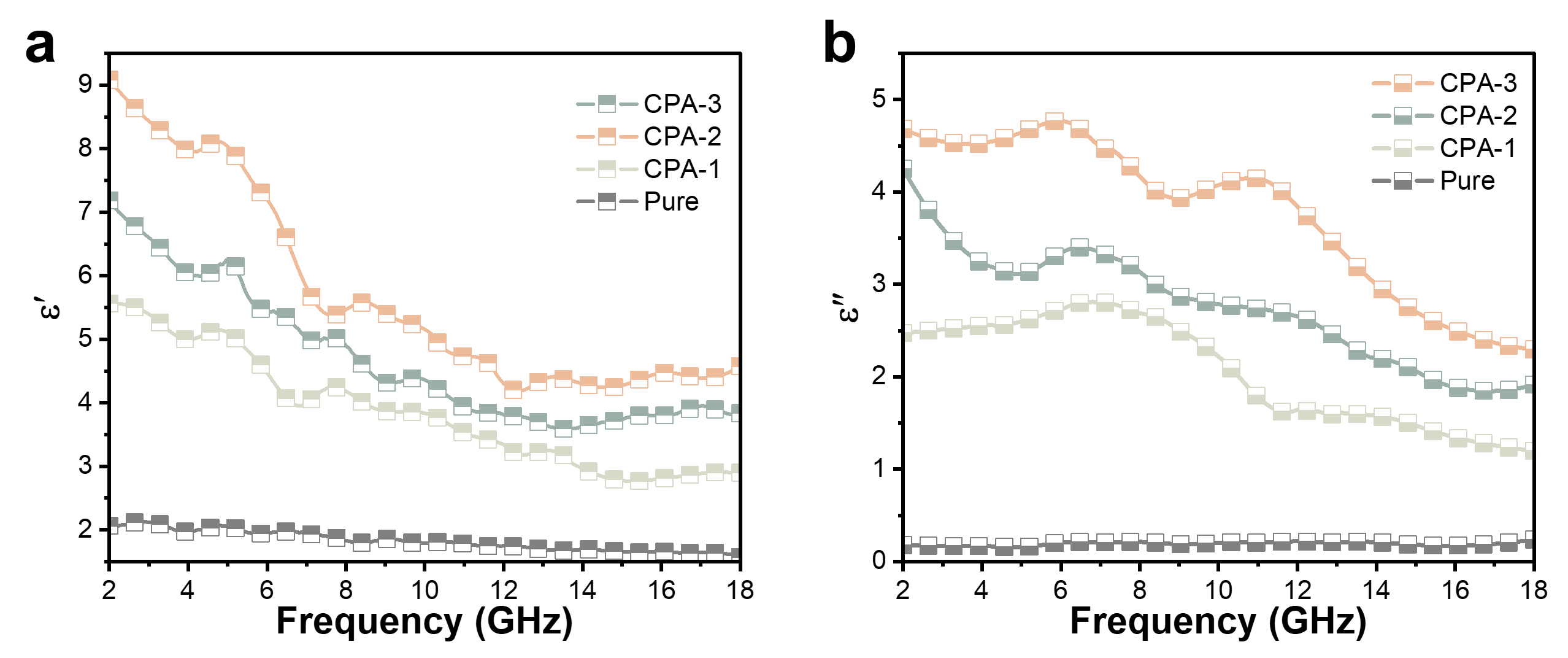


**Fig. S14.** The (a) *ε′* and (b)*ε′′* values of the pure PI and CPA.


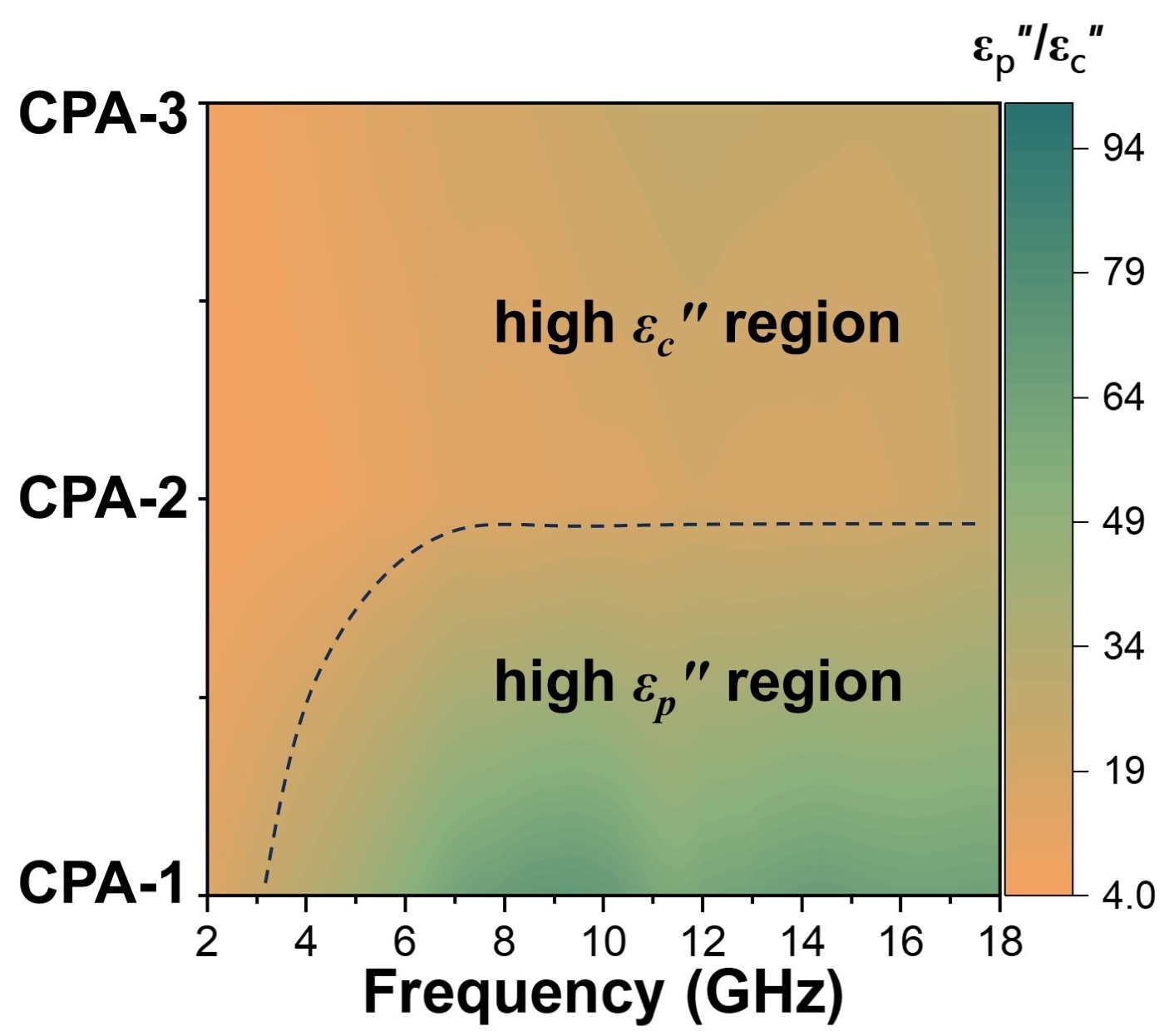


**Fig. S15.** Distribution of the *ε_p_′′/ε_c_′′* ratio for the CPA.


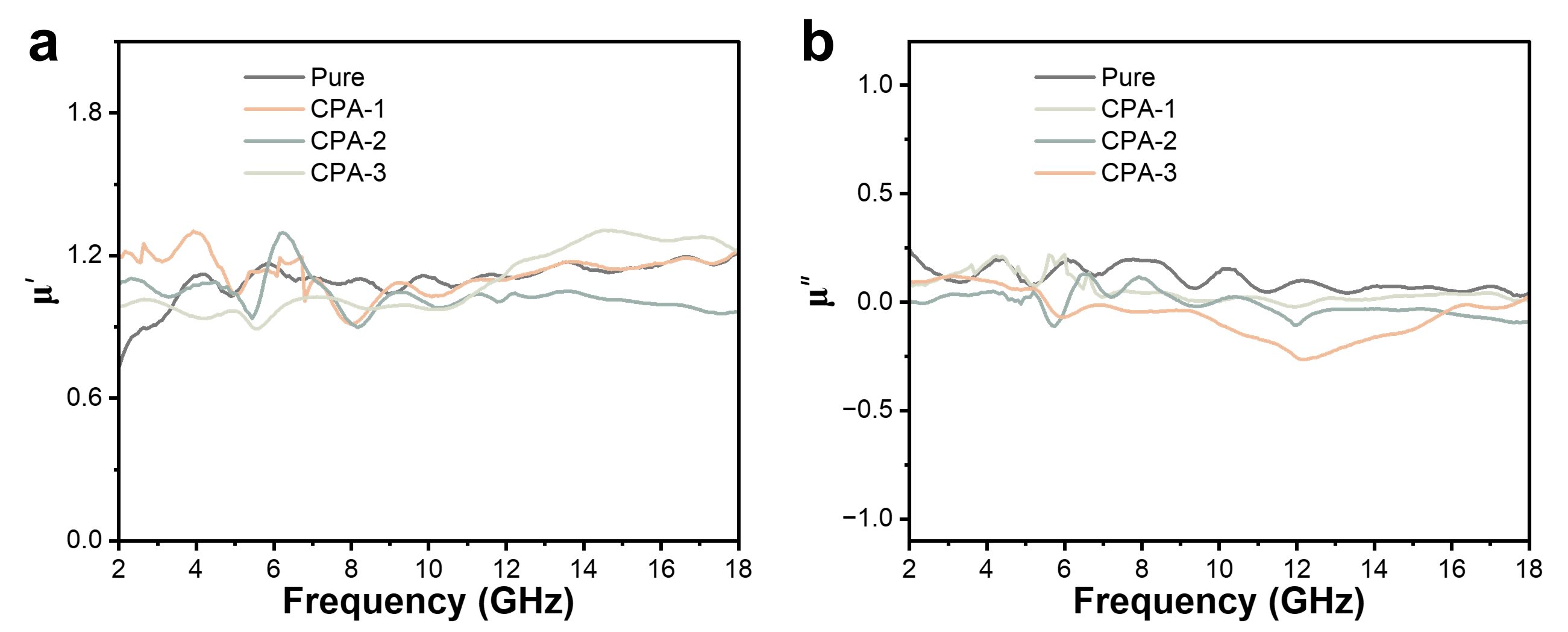


**Fig. S16.** The (a) *μ′* and (b) *μ′′* values of the pure PI and CPA.


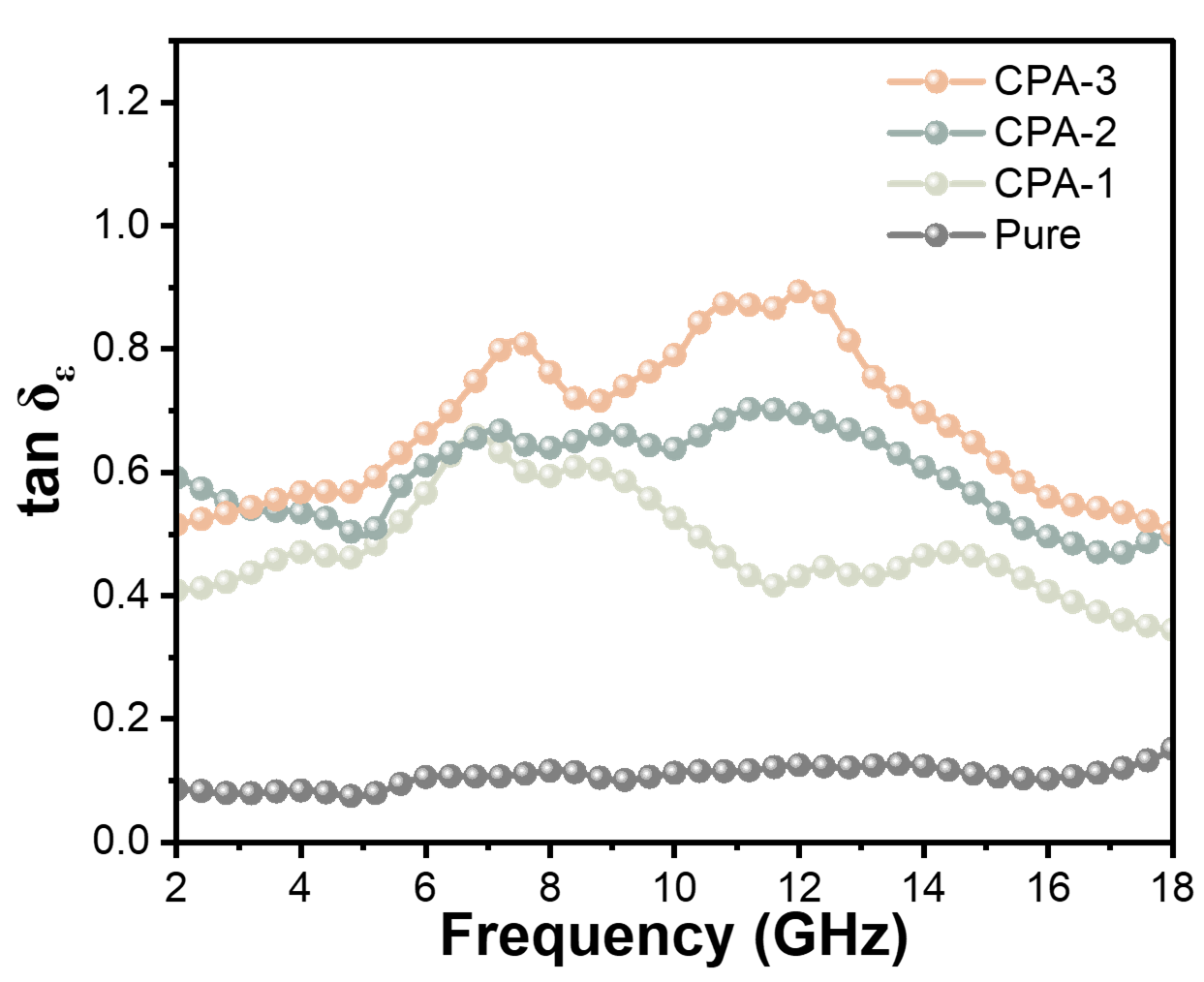


**Fig. S17.** The *tan δ_ε_* values of the pure PI and CPA.


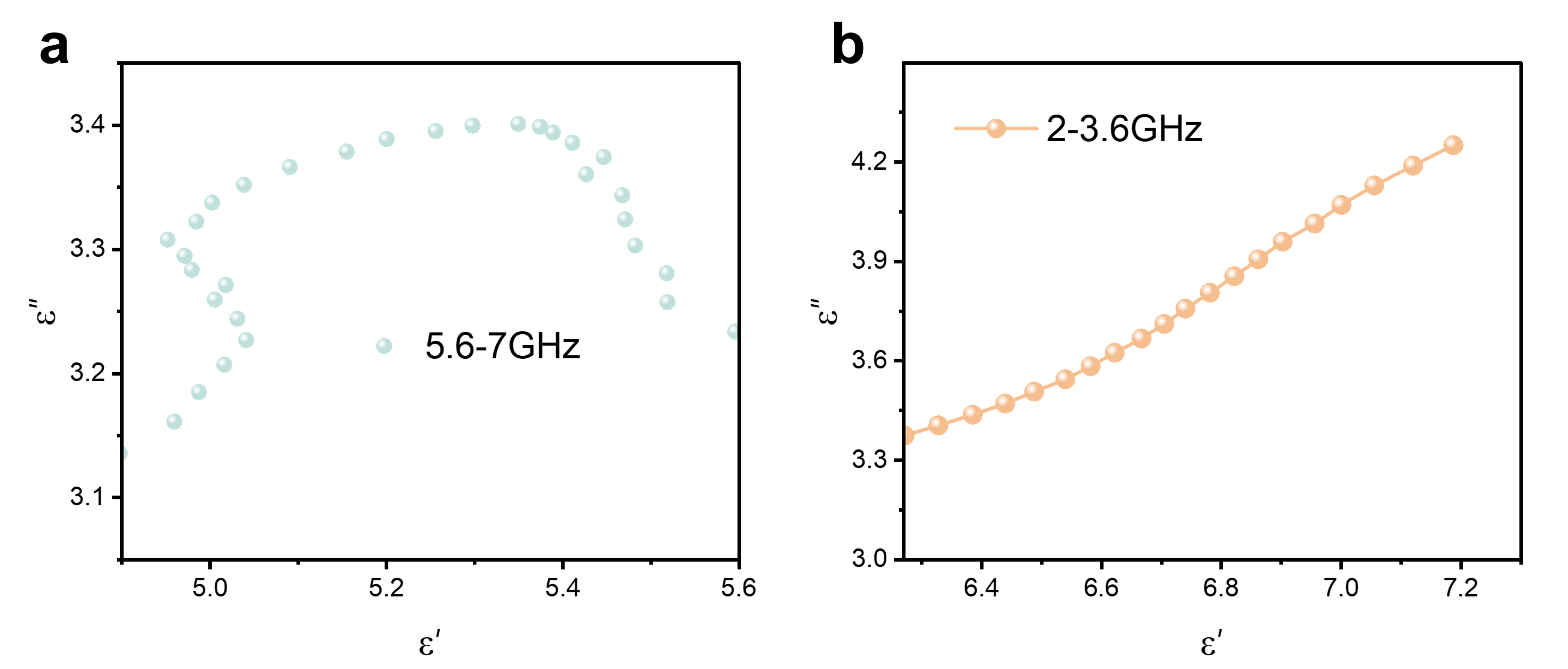


**Fig. S18.** The Cole-Cole curves of CPA-2.


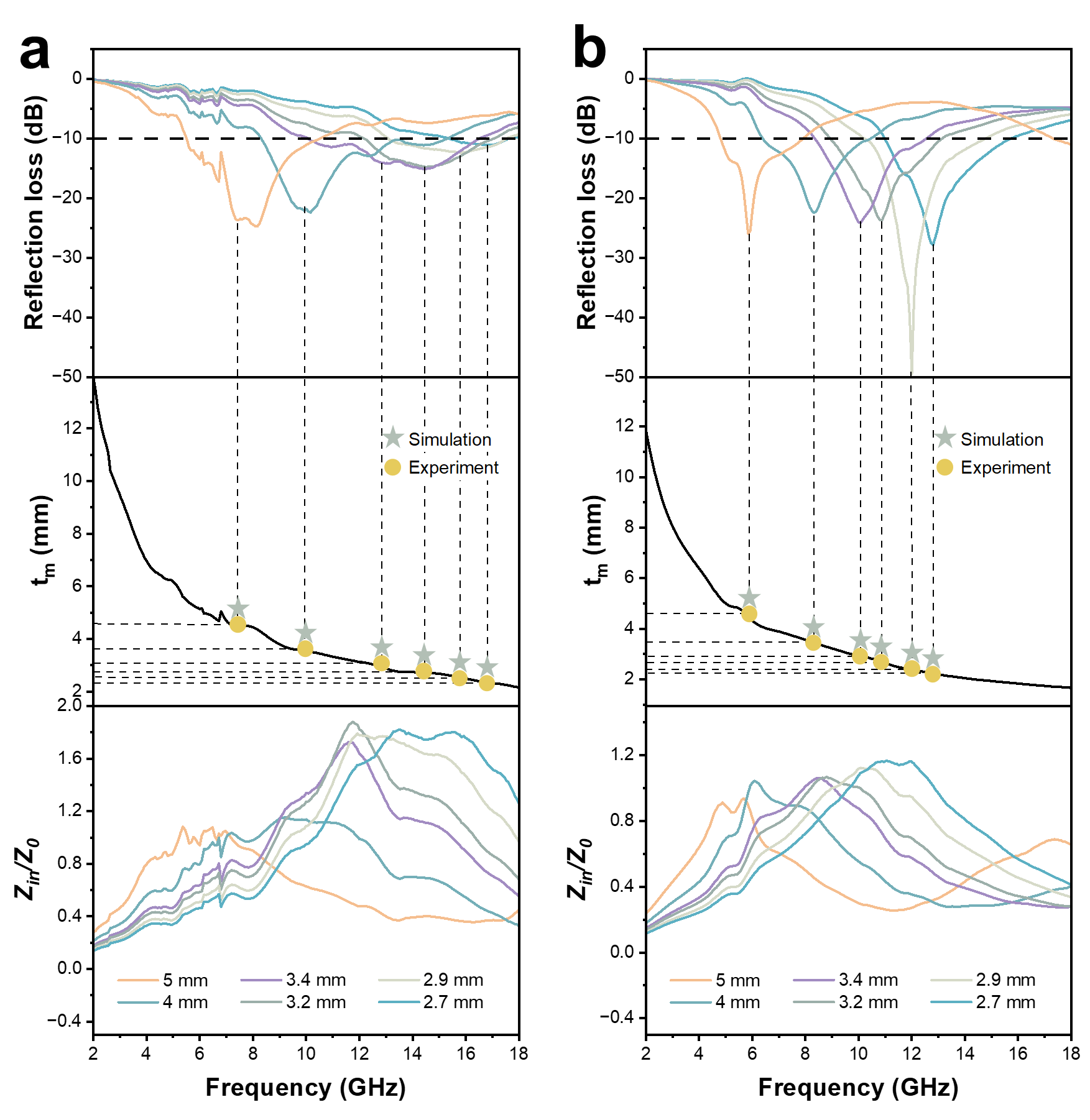


**Fig. S19.** The RL, dependence of matched thickness (*t_m_*) on matched frequency (*f_m_)* at *λ*/4 wavelength, and impedance-matching characteristics (*Z* = *Z_in_*/*Z_0_*) of (a) CPA-1 and (b) CPA-3.


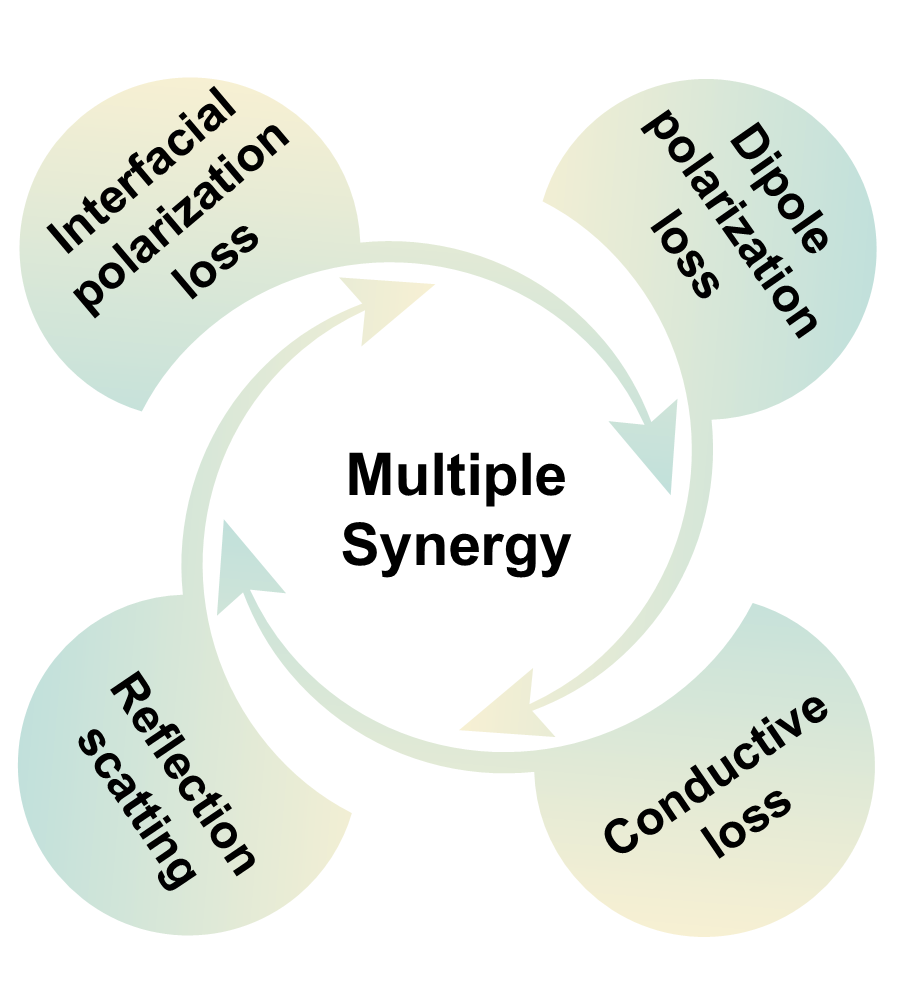


**Fig. S20.** Scheme of primary microwaves attenuation process in the CPA.


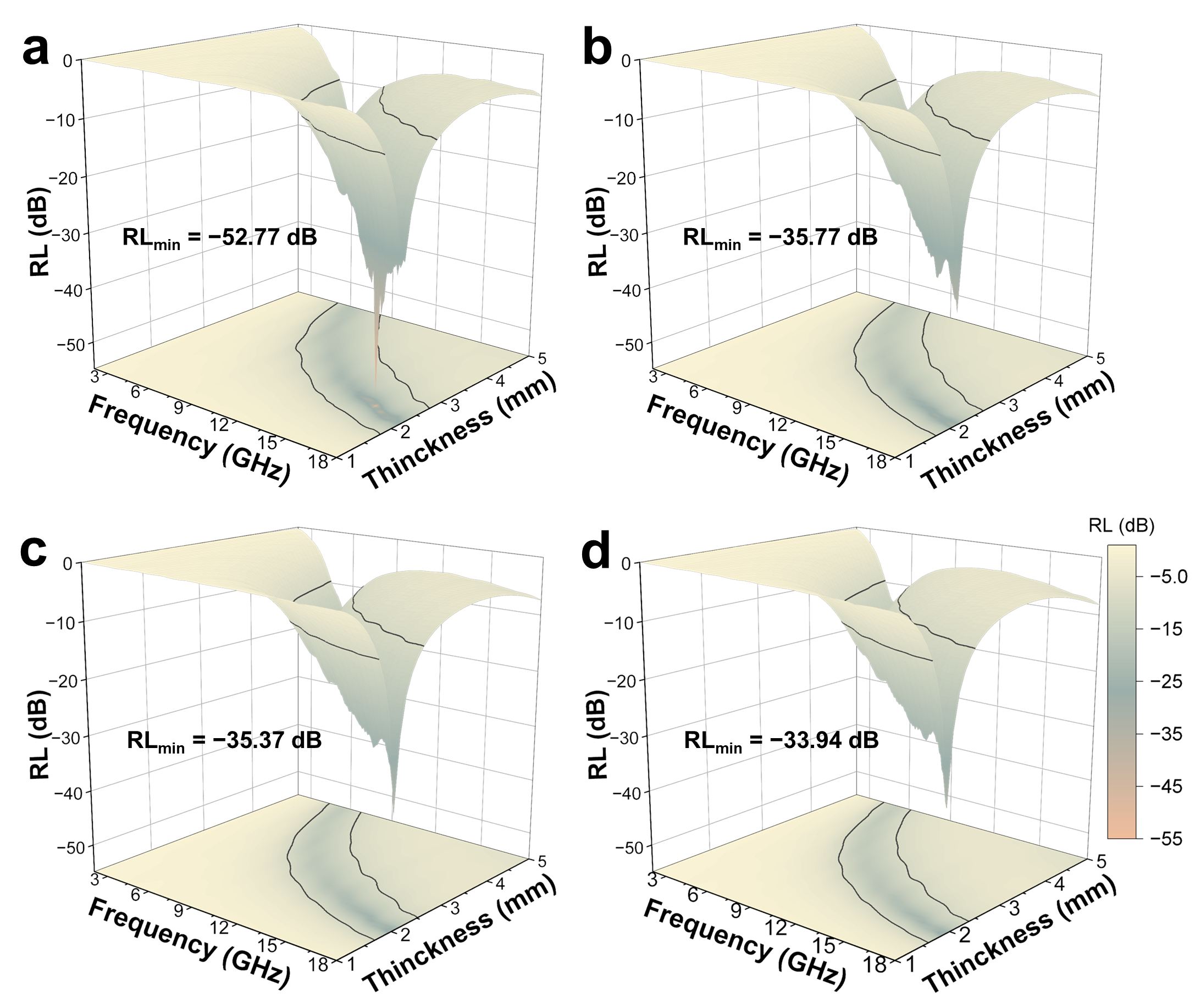


**Fig. S21.** 3D RL diagrams of CPA-2 under (a) 10%, (b) 20%, (c) 30%, and (d) 50% compressive ratios.


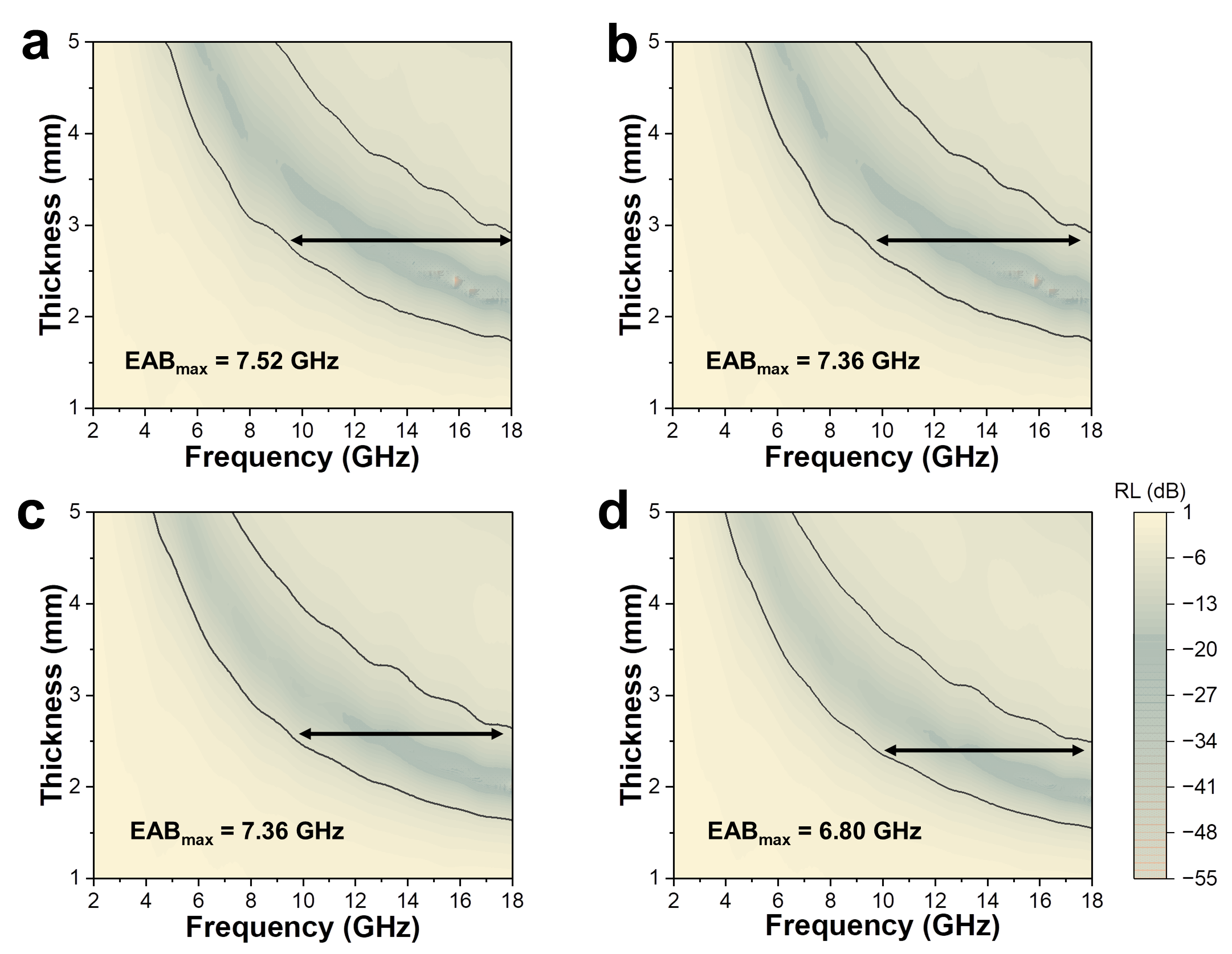


**Fig. S22.** 2D RL diagrams of CPA-2 under (a) 10%, (b) 20%, (c) 30%, and (d) 50% compressive ratios.


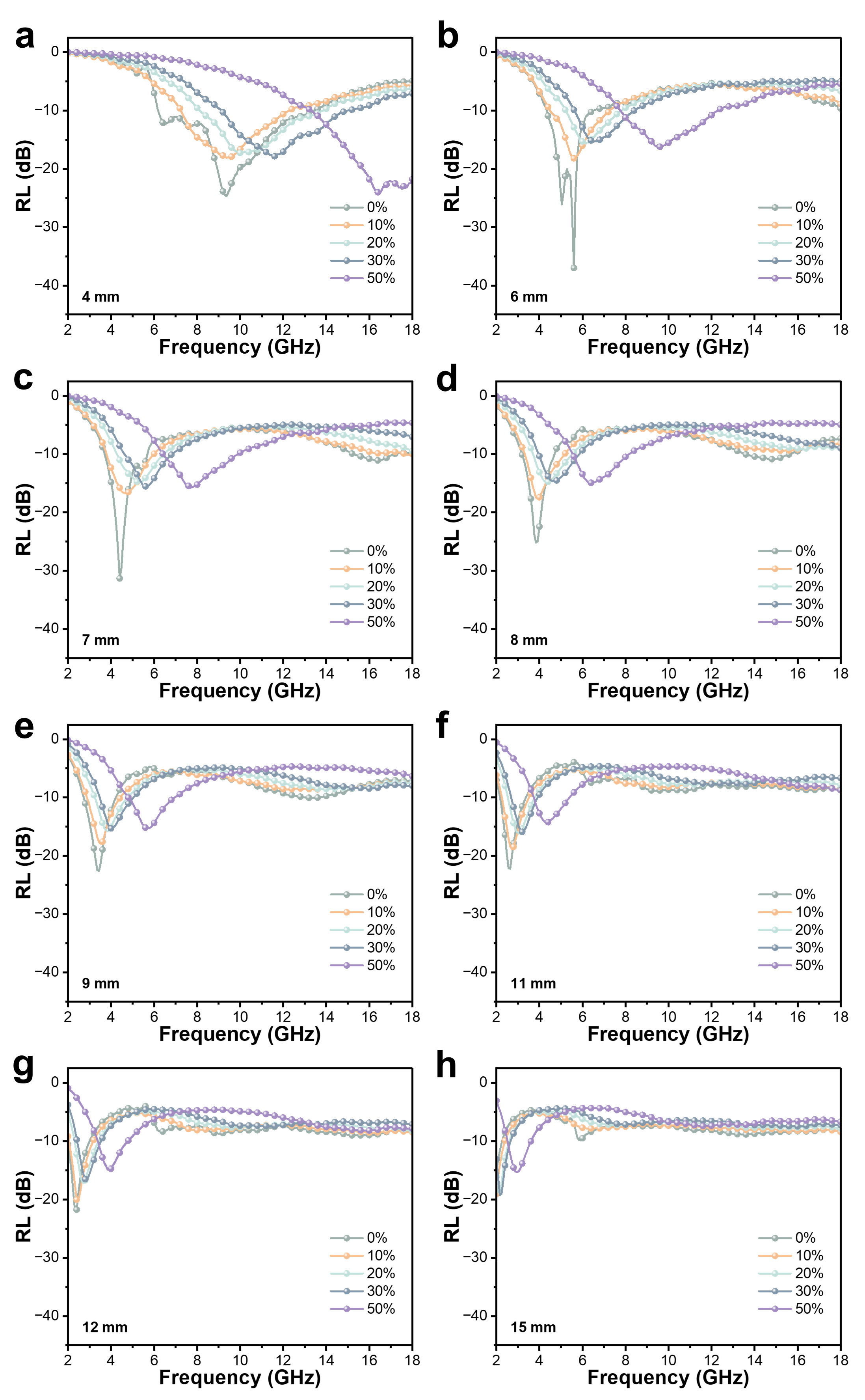


**Fig. S23.** The RL values of CPA-2 under different compression ratios and different initial thicknesses.


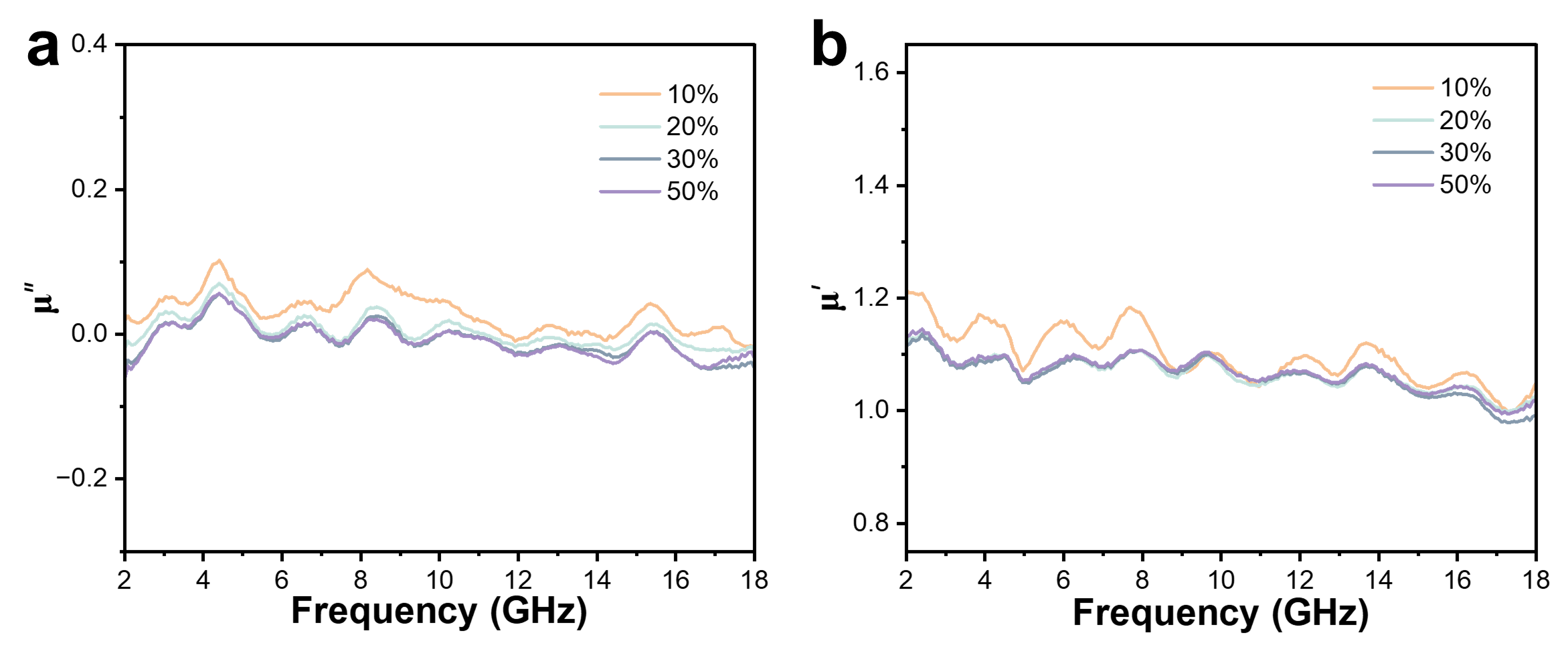


**Fig. S24.** The (a) *μ′* and (b) *μ′′* values of the CPA-2 under different compression ratios.


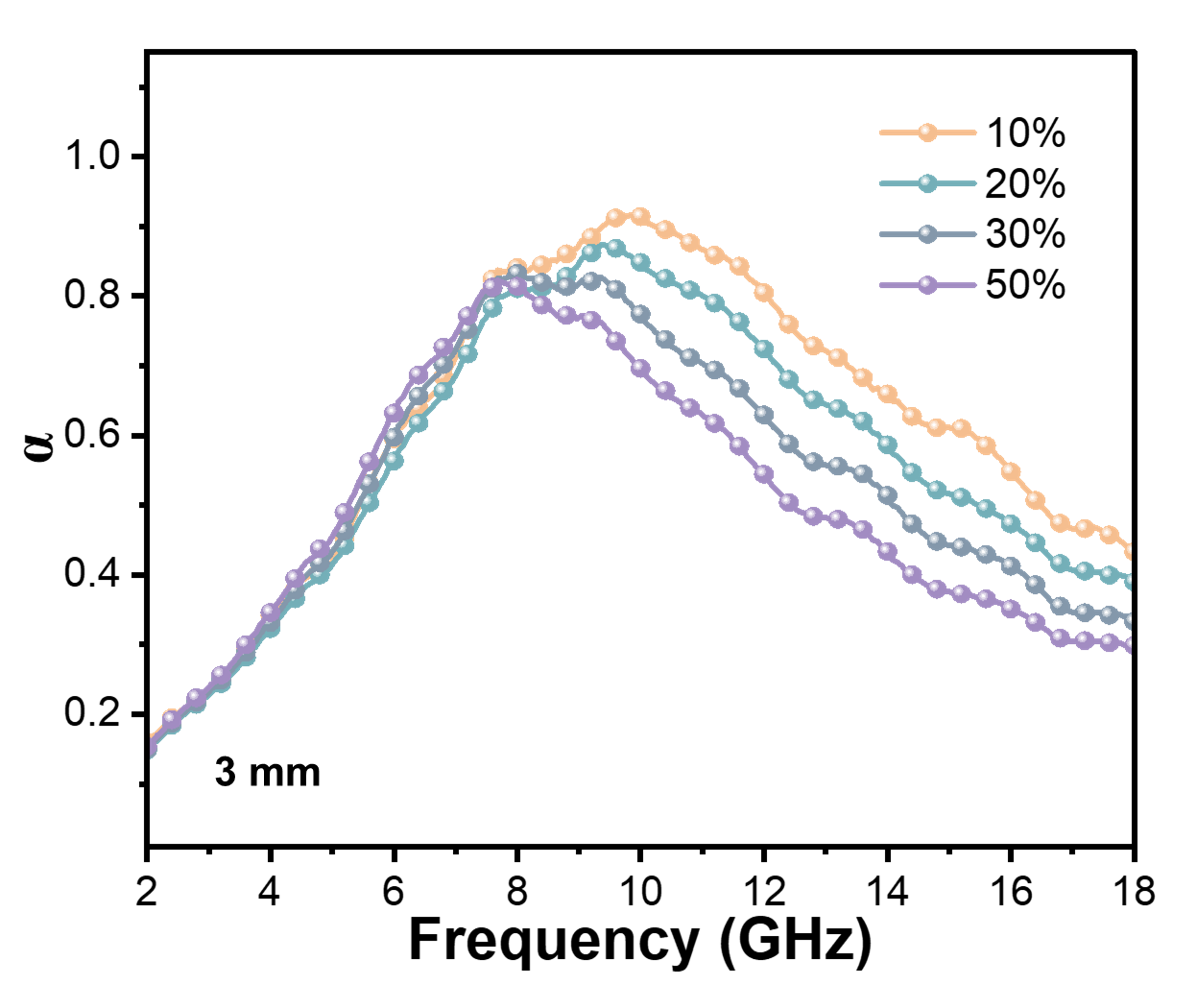


**Fig. S25.** The *α* of CPA-2 under different compressive ratios with a thickness of 3 mm.


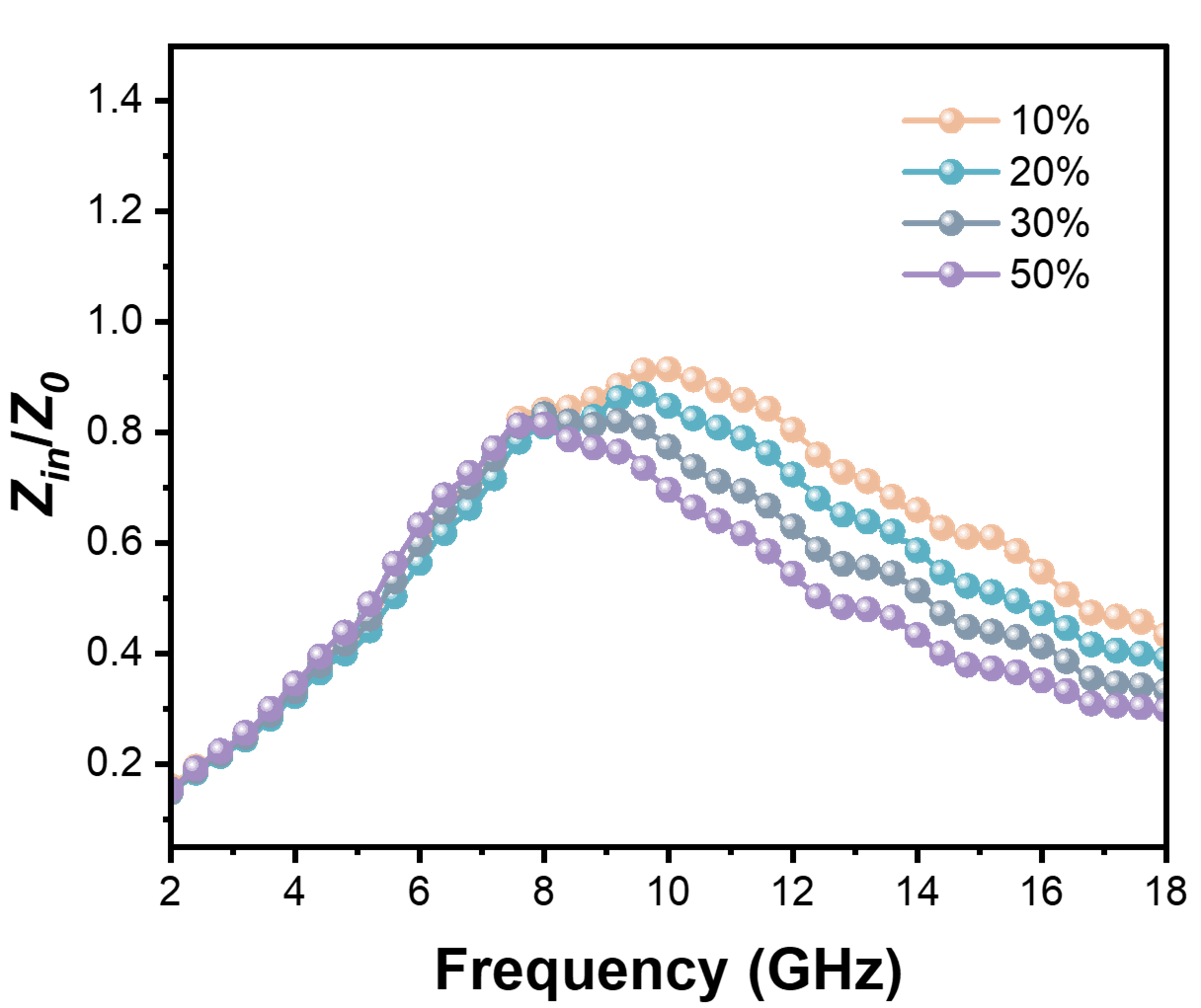


**Fig. S26.** The *Z_in_/Z_0_* values of CPA-2 under different compressive ratios with a thickness of 3 mm.


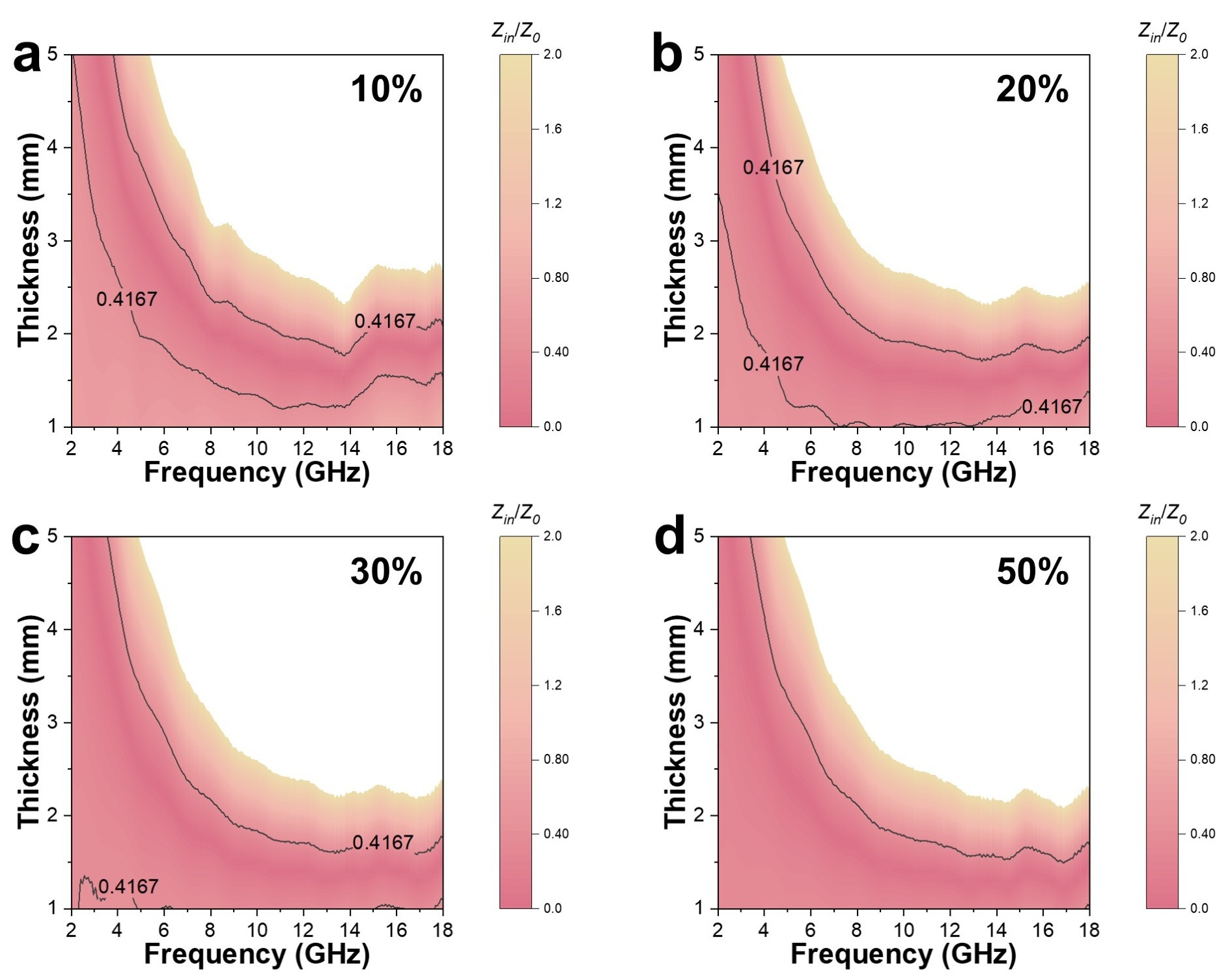


**Fig. S27.** (a−d) The delta (*Δ,* 0 ≤ |*Z_in_*/*Z_0_* − 1| ≤ 0.2) function corresponding to the CPA-2 under different compression ratios.


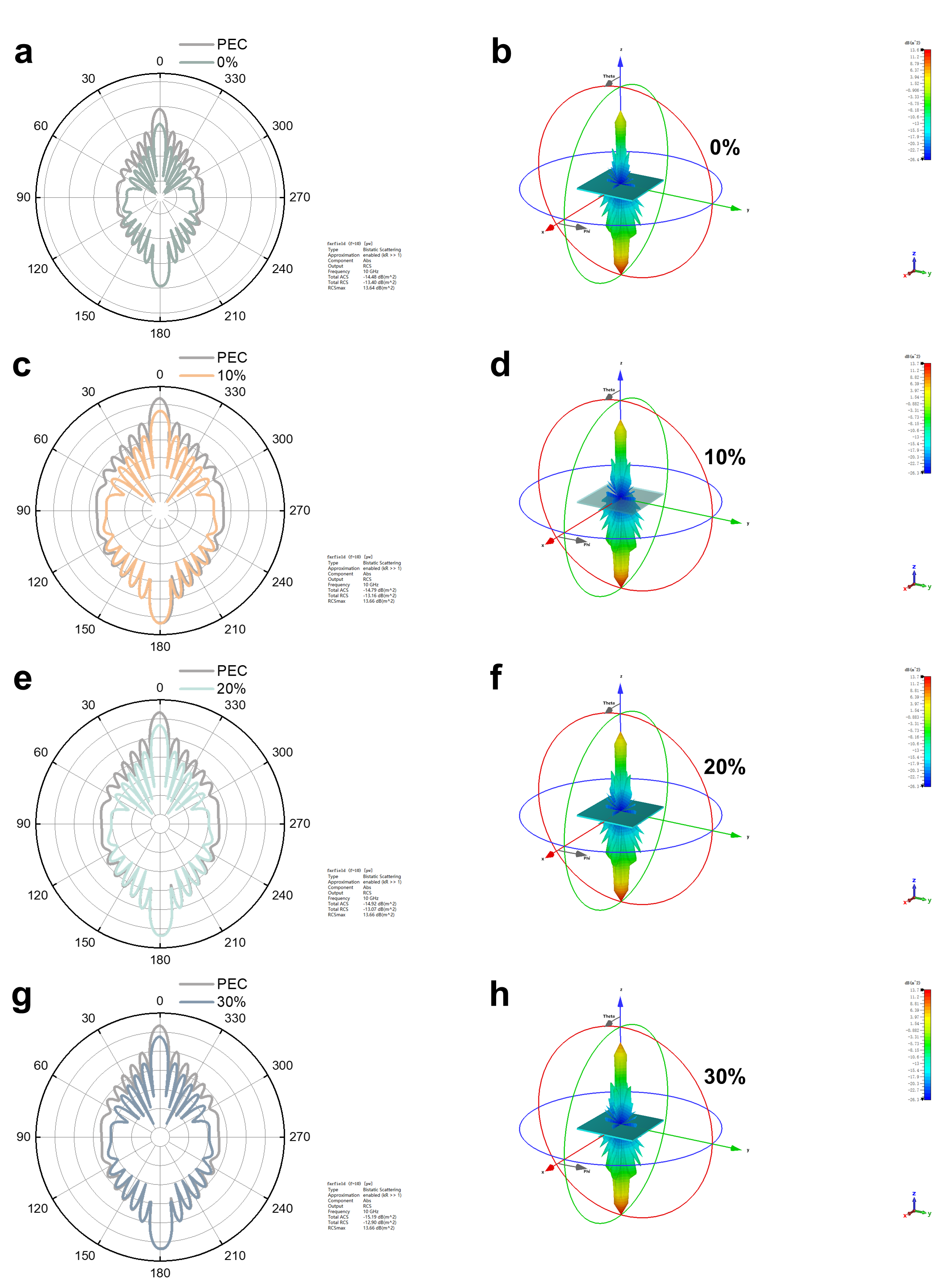


**Fig. S28.** The RCS curves and 3D RCS plots of CPA-2 under different compression ratios.


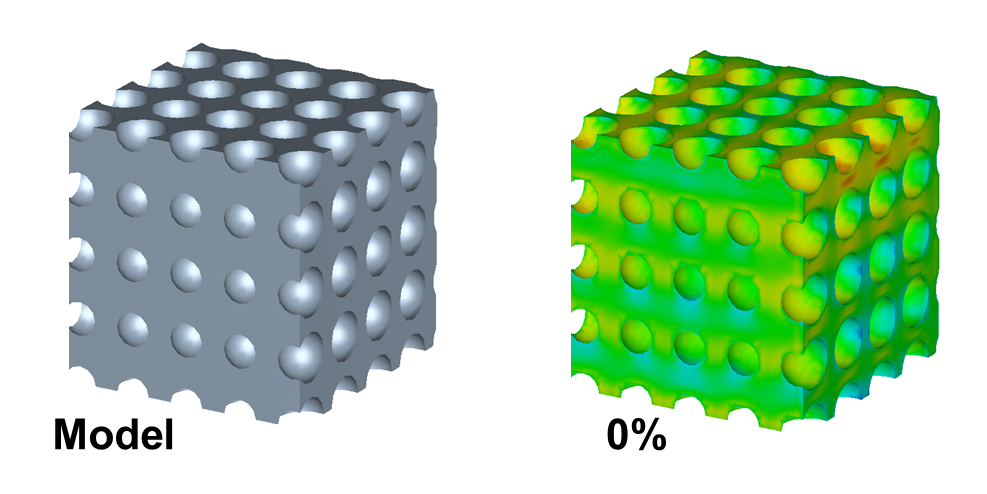


**Fig. S29.** The 3D power loss density plots of CPA-2 under different compression ratios.


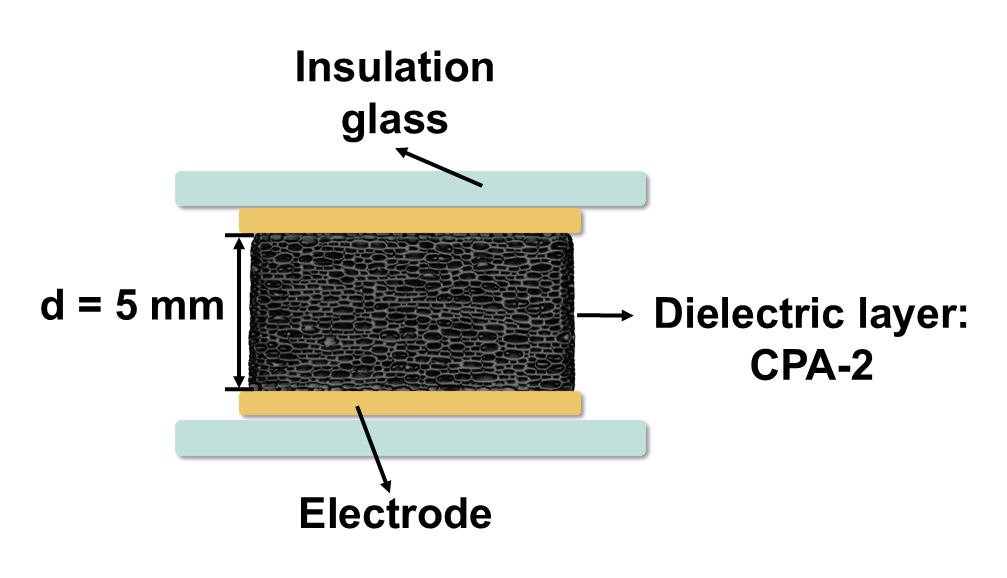


**Fig. S30.** The capacitance test model of CPA.


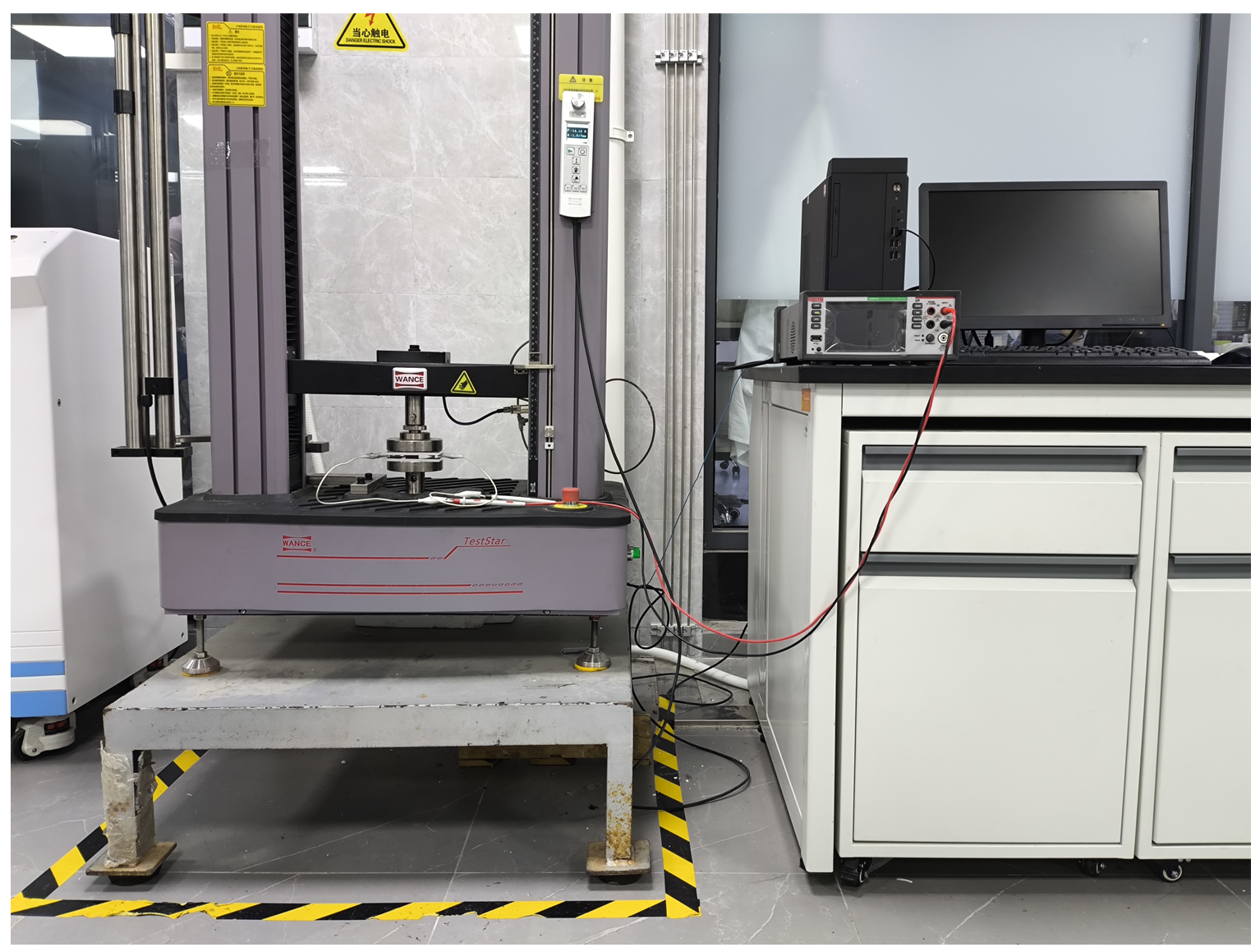


**Fig. S31.** The established capacitance testing device.


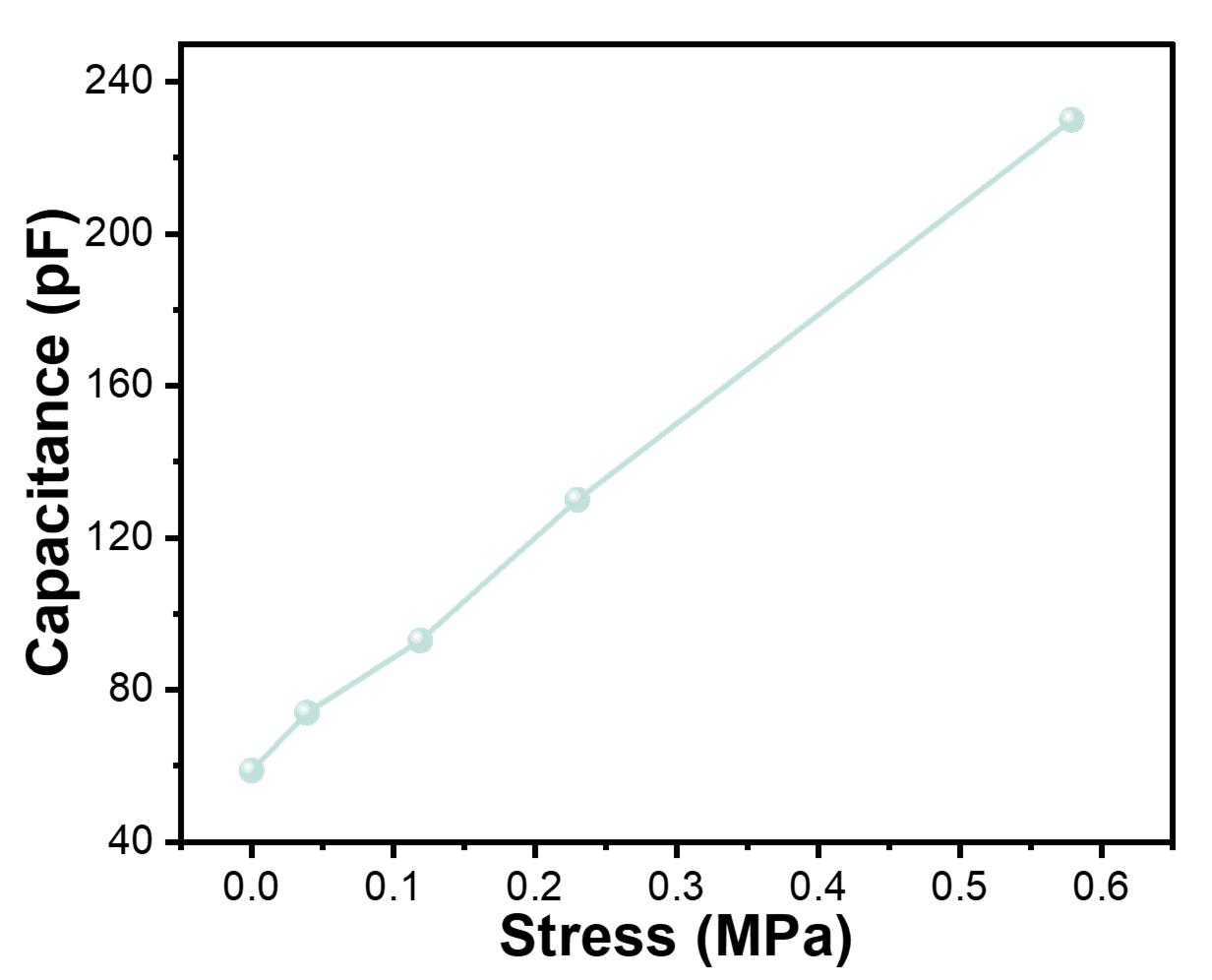


**Fig. S32.** The capacitance values of CPA-2 under different compression ratios.


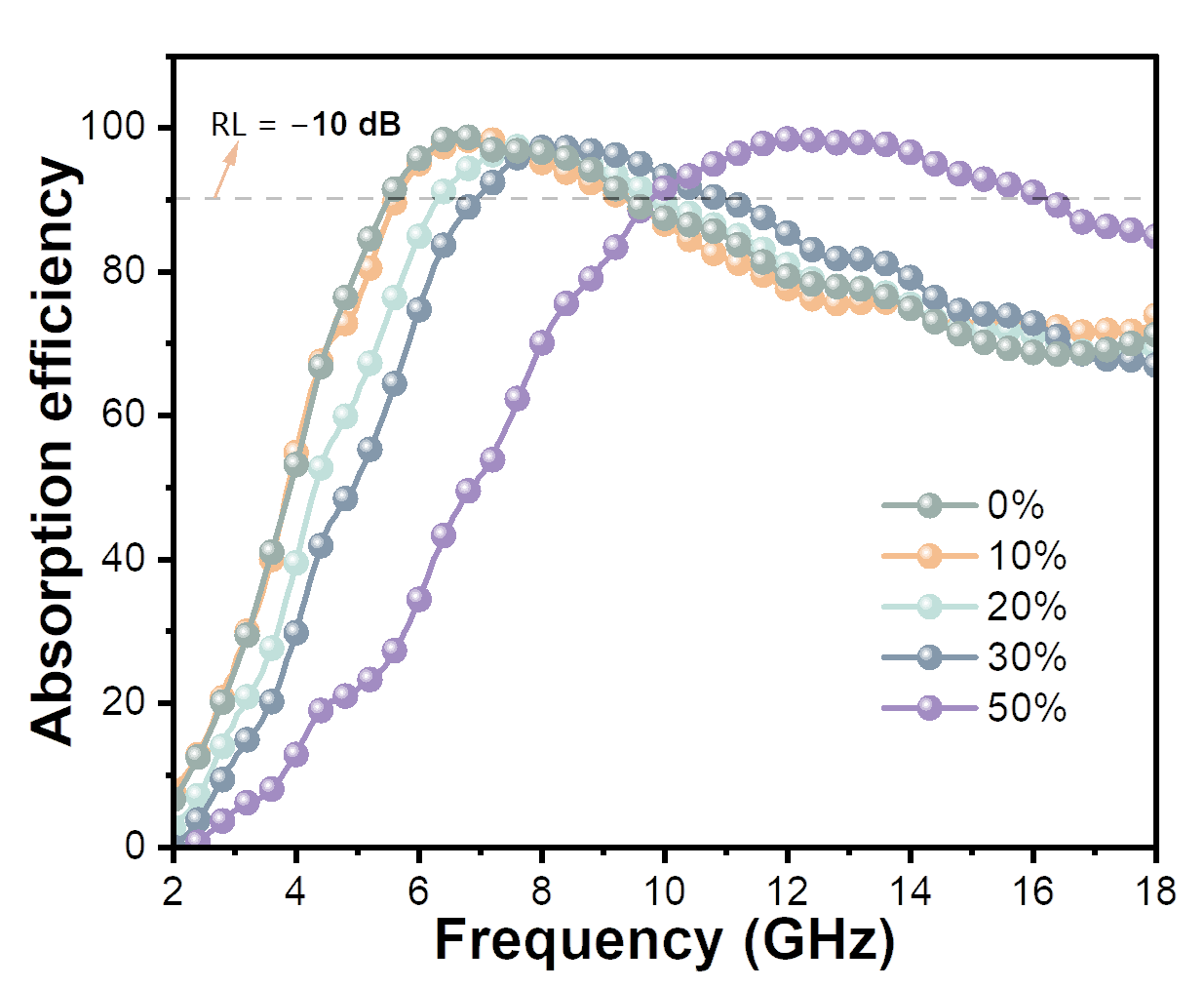


**Fig. S33.** The corresponding absorption efficiencies of the CPA-2 under different compression ratios.
